# Medical disaster response: A critical analysis of the 2010 Haiti earthquake

**DOI:** 10.3389/fpubh.2022.995595

**Published:** 2022-11-01

**Authors:** Matthew Keith Charalambos Arnaouti, Gabrielle Cahill, Michael David Baird, Laëlle Mangurat, Rachel Harris, Louidort Pierre Philippe Edme, Michelle Nyah Joseph, Tamara Worlton, Sylvio Augustin, The Haiti Disaster Response – Junior Research Collaborative (HDR-JRC)

**Affiliations:** ^1^Department of Global Health and Social Medicine, Harvard Medical School, Boston, MA, United States; ^2^Program in Global Surgery and Social Change, Harvard Medical School, Boston, MA, United States; ^3^Department of Orthopaedic Surgery, Walter Reed National Military Medical Center, Bethesda, MD, United States; ^4^Faculté de Médecine et de Pharmacie de l'Université d'État d'Haïti, Port-au-Prince, Haiti; ^5^Department of Surgery, Uniformed Services University, Bethesda, MD, United States; ^6^Hôpital La Providence des Gonaïves, Gonaïves, Haiti; ^7^Clinical Trials Unit, University of Warwick, Warickshire, United Kingdom; ^8^Hôpital de l'Universite d'Etat d'Haïti, Port-au-Prince, Haiti

**Keywords:** humanitarian response, disaster response, military humanitarianism, Haiti earthquake, United Nations disaster response

## Abstract

**Introduction:**

On January 12, 2010, a 7.0 magnitude earthquake struck the Republic of Haiti. The human cost was enormous—an estimated 316,000 people were killed, and a further 300,000 were injured. The scope of the disaster was matched by the scope of the response, which remains the largest multinational humanitarian response to date. An extensive scoping review of the relevant literature was undertaken, to identify studies that discussed the civilian and military disaster relief efforts. The aim was to highlight the key-lessons learned, that can be applied to future disaster response practise.

**Methods:**

Preferred Reporting Items for Systematic reviews and Meta-Analyses extension for Scoping Reviews guidance was followed. Seven scientific databases were searched, using consistent search terms—followed by an analysis of the existent Haitian literature. This process was supplemented by reviewing available grey literature. A total of 2,671 articles were reviewed, 106 of which were included in the study. In-depth analysis was structured, by aligning data to 12 key-domains, whilst also considering cross-sector interaction (Civilian-Civilian, Military-Military, and Civilian-Military). Dominant themes and lessons learned were identified and recorded in an online spreadsheet by an international research team. This study focuses on explicitly analysing the medical aspects of the humanitarian response.

**Results:**

An unpreceded collaborative effort between non-governmental organisations, international militaries, and local stakeholders, led to a substantial number of disaster victims receiving life and limb-saving care. However, the response was not faultless. Relief efforts were complicated by large influxes of inexperienced actors, inadequate preliminary needs assessments, a lack of pre-existing policy regarding conduct and inter-agency collaboration, and limited consideration of post-disaster redevelopment during initial planning. Furthermore, one critical theme that bridged all aspects of the disaster response, was the failure of the international community to ensure Haitian involvement.

**Conclusions:**

No modern disaster has yet been as devastating as the 2010 Haiti earthquake. Given the ongoing climate crisis, as well as the risks posed by armed conflict—this will not remain the case indefinitely. This systematic analysis of the combined civilian and military disaster response, offers vital evidence for informing future medical relief efforts—and provides considerable opportunity to advance knowledge pertaining to disaster response.

## Introduction

The Republic of Haiti^1^ is the first nation state to be founded by former slaves (3), after gaining independence from colonial rule in 1804 (2). Its history has been tumultuous—the nation has been marred by political instability, a number of *coups d'état*, dictatorial regimes, and international interventions and occupations (2). This, in addition to the imposition of neo-liberal economic and development policy, has resulted in economic fragility and drastic demographic alterations, over the course of Haiti's maturation as a sovereign state (1). The Haitian population has largely gravitated towards major cities, which have become increasingly congested—particularly the nation's capital, Port-au-Prince (1). To facilitate such increases in population density, significant developments in housing have been required—with efforts widely failing to adhere to safe standards of construction (1). Furthermore, the poverty rate within Haiti has increased from 50 to 80% (1, 2). Currently, Haiti has the lowest Gross Domestic Product (GDP) per capita in the Latin American and Caribbean region (4), and the 30th lowest GDP per capita on purchasing power parity, globally (5).

On January 12th, 2010, a 7.0-magnitude earthquake struck Haiti. Its epicentre was just 15.5 miles from the capital, Port-au-Prince (6). The earthquake, and the 52 significant aftershocks^2^ that followed, were catastrophic (6). The human cost was enormous; as many as 316,000^3^ people were killed, 300,000 more were injured, 2 million were displaced, and a total of 3 million were directly affected (7, 10–12). For Haiti, an already vulnerable state, this disaster was a “worst-case” scenario. The nation lost key government capacity and leadership, with both political and primary security force leaders being killed by the earthquake (12). It also lost function of its electricity grid, telecommunications network, air, and seaports (13). The earthquake caused extensive damage to Haiti's already limited infrastructure and response capability (6). Healthcare services were particularly vulnerable, given that prior to the disaster, 47% of Haitians lacked access to even basic medical care, and external organisations provided 75% of the nation's healthcare (14). Thirty of the forty-nine medical facilities, within the regions impacted by the earthquake, were either partially or completely destroyed (15)—including, the only national tertiary care centre (6). The combination of substantial structural damage, and the large numbers of traumatically injured earthquake victims, meant that the local health system was at extreme risk of being overwhelmed.

The international community, responded to this need *en masse*, mounting one of the largest humanitarian relief efforts to date (16). Assistance arrived rapidly, in large numbers, and with varying levels of capacity and skill (11). A multitude of actors offered assistance, including both civilian and military organisations (2). With so many different agencies being involved, it is clear that coordination and communication during relief efforts, was required. When armed forces are involved in a response, coordination can be divided into three categories: Civilian-Civilian, Civilian-Military, and Military-Military. In the context of this study, Civilian refers to any non-military actors—such as government agencies, United Nations (UN) organisations, and Non-Governmental Organisations (NGO). The UN states that “essential dialogue and interaction between civilian and military actors in humanitarian emergencies… is necessary to protect and promote humanitarian principles, avoid competition, minimise inconsistency, and when appropriate, pursue common goals” [(17), Paragraph 1].

This scoping review seeks to analyse the medical component of the complex international, multi-sector response—identifying dominant themes within relevant literature, as well as highlighting the key lessons learned. Particular emphasis has been placed on the interaction between civilian and military actors involved in medical relief efforts, with the aim of informing guidelines that can improve collaborative efforts in future disaster responses, and direct future research.

## Methodology

Utilising library scientists, an extensive scoping review of the relevant literature was undertaken. This process was designed to be reproducible, and articles were gathered through conducting verified, systematic searches of seven scientific databases (PubMed, Medline, World of Science, Embase, CINAHL, PsycInfo, Google Scholar)—utilising consistent search terms (Table 1). Preferred Reporting Items for Systematic reviews and Meta-Analyses extension for Scoping Reviews (PRISMA-ScR) guidelines were followed (18). The review was undertaken between June 14th 2020 and October 4th 2021. The screening process was conducted, using Covidence systematic review screening software (https://www.covidence.org/, Veritas Health Innovation, Melbourne, Australia).

**Table 1:** Search terms utilised.

Haiti^*^	Earthquake^*^
Disaster	Response
Plan	Management
Preparedness	Recovery
Relief	Risk
Emergency	Military
Military medicine	Humanitarian
International cooperation	After action
Disaster planning	Emergency health service
Surge capacity	Medical countermeasure

* These were required search terms. The remaining terms were utilised in conjunction with these core terms—in various combinations.

To establish the search terms (Table 1), two preliminary tasks were undertaken.

1) Structured interviews with:
  a. Senior Haitian civilian clinicians
    i. Dr. Louis-Franck Télémaque^4^.
    ii. Dr. Frédéric Barau Déjean^5^.
  b. United States (US) military personnel
    i. Professor David Polatty^6^.
    ii. Captain Andrew Johnson^7^.
2) Review of two key-reports, analysing the earthquake response:
  a. *Response to the Humanitarian Crisis in Haiti Following the 12 January 2010 Earthquake: Achievements, Challenges and Lessons to Be Learned* (6).
  b. *The U.S. Military Response to the 2010 Haiti Earthquake: Considerations for Army Leaders* (12).

This process enabled the identification of key-domains of analysis, for establishing the lessons learned during the disaster response. The following eligibility criteria, were designed to ensure adequate data capture from the multiple entities and non-academic institutions, that were substantially involved in the earthquake response—but have historically disseminated reports outside of the traditional peer-review process. Twelve domains were recognised as relevant: Humanitarian and Military Response, Communication, Coordination, Resources, Needs Assessment, Pre-Existing Policy, Workforce/Infrastructure Loss, Timeliness/Timing of Response, Expertise, Military/Political Interaction/Conflict, External and Unknown Factors, and Preventable Deaths. Inclusion criteria mirrored these, and literature was to be included if information corresponding to one or more of the key-domains was identified. Exclusion criteria were: if there was no information on civilian-military response; if the article was not focused on the earthquake response; if there was an overly clinical focus^8^; if the article focused on long-term recovery without discussing relief efforts; if the article was a duplicate; if the full-text was unavailable; or if the article was published before January 12th 2010.

An initial 2,336 studies were identified from the database searches, 511 of which were immediately excluded as duplicates. Following abstract screening, with each title and abstract screened by two members of the study team, an additional 1,697 articles were excluded. A subsequent full-text review was undertaken, with each document being reviewed by two study team members, for inclusion or exclusion. A further 73 articles were identified as ineligible during this stage of the review—the full-text of one article was irretrievable, and so this was also excluded. The Haitian literature was also assessed, in its entirety, for all articles related to the earthquake response. The initial search, for any studies related to earthquakes in Haiti, identified 272 articles. After full-text review, three articles were found to be related to the 2010 response, and were included.

This process was supplemented by grey literature reviews, to identify unclassified military documents for inclusion in the study. At this stage, some articles with an exclusively civilian focus were included for review. A further 58 articles were identified during this process, four of which were noted to be ineligible for inclusion in the study.

The reference lists of included articles were reviewed (backward snowballing), to determine if any cited works were eligible for inclusion—five additional studies were identified, four of which were included. Finally, citations of included articles were searched, to identify any relevant studies that had cited them (forward snowballing)—although, no further studies were included in this manner.

**Figure 1:**
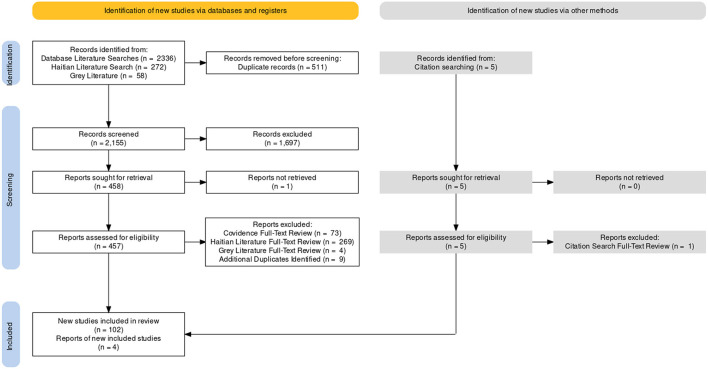
Preferred reporting items for systematic reviews and meta-analyses (PRISMA) flow diagram. A total of 106 articles were included. This flow diagram was created, using Evidence Synthesis Hackathon software (https://www.eshackathon.org/, Evidence Synthesis Hackathon).

**Figure 2:**
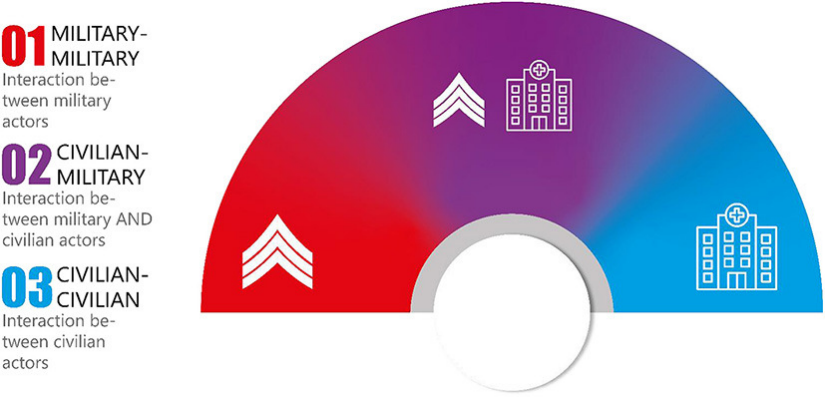
Sector interaction.

**Figure 3:**
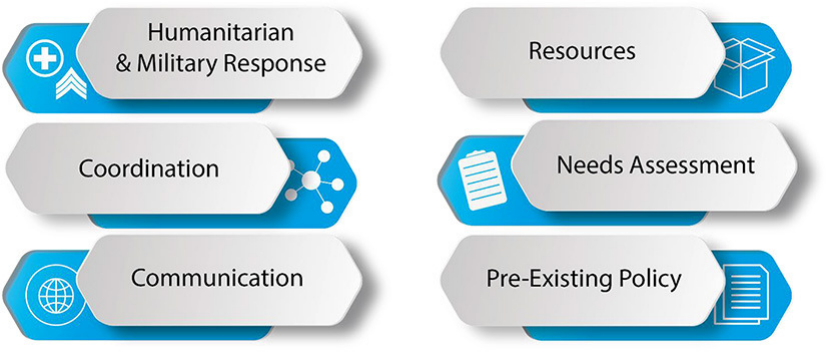
Priority domains.

Nine additional studies were noted to be duplicates during the extraction process, and were subsequently excluded. The final number of articles, from which data was extracted, was 106 (Figure 1; Tables 2–5). In-depth analysis was structured by aligning data pertaining to the aforementioned 12 key-domains^9^, and by sector-interaction (Civilian–Civilian, Military–Military, and Civilian–Military) (Figure 2). Dominant themes and lessons learned were identified and recorded, in an online table, by the ten reviewers. This data was then synthesised, and further examined, to focus more explicitly on medical elements of the response. This study will focus on the analysis of priority domains, the first 6 key-domains listed, as determined by the principal investigators (MJ and TW) (Figure 3).

**Table 2:** Database searches: articles included.

**Title**	**Author(s)**	**Source**
1 Canadian Field Hospital in Haiti: Surgical Experience in Earthquake Relief	Talbot, M., Meunier, B., Trottier, V., Christian, M., Hillier, T., Berger, C., McAlister, V., and Taylor, S.	Talbot, M., Meunier, B., Trottier, V., Christian, M., Hillier, T., Berger, C., et al. 1 Canadian Field Hospital in Haiti: Surgical Experience in Earthquake Relief. *Canadian Journal of Surgery*. 2012; 55(4): 271-274. Available at: https://dx.doi.org/10.1503/cjs.039010
A Call To Respond: The International Community's Obligation To Mitigate the Impact of Natural Disasters	Hernandez, J. R. and Johnson, A. D.	Hernandez, J. R., Johnson, A. D. A Call to Respond: The International Community's Obligation to Mitigate the Impact of Natural Disasters. *Emory International Law Review*. 2011; 25(3): 1087-1096. Available at: https://scholarlycommons.law.emory.edu/eilr/vol25/iss3/2
Actorness and Effectiveness in International Disaster Relief: The European Union and United States in Comparative Perspective	Brattberg, E. and Rhinard, M.	Brattberg, E., Rhinard, M. Actorness and Effectiveness in International Disaster Relief: The European Union and United States in Comparative Perspective. *International Relations*. 2013; 27(3): 356-374. Available at: https://dx.doi.org/10.1177/0047117813497298
Air Force Disaster Response: Haiti Experience	Stuart, J. J. and Johnson, D. C.	Stuart, J. J., Johnson, D. C. Air Force Disaster Response: Haiti Experience. *Journal of Orthopaedic Surgical Advances*. 2011; 20(1): 62-66. Available at: https://pubmed.ncbi.nlm.nih.gov/21477536/
Analysis of the International and US Response to the Haiti Earthquake: Recommendations for Change	Kirsch, T., Sauer, L., and Guha-Sapir, D.	Kirsch, T., Sauer, L., Guha-Sapir, D. Analysis of the International and US Response to the Haiti Earthquake: Recommendations for Change. *Disaster Medicine and Public Health Preparedness*. 2012; 6(3): 200-208. Available at: https://dx.doi.org/10.1001/dmp.2012.48
Application of Health Technology in Humanitarian Response: U.S. Military Deployed Health Technology Summit—A Summary	Doarn, C. R., Barrigan, C. R., and Poropatich, R. K.	Doarn, C. R., Barrigan, C. R., Poropatich, R. K. Application of Health Technology in Humanitarian Response: U.S. Military Deployed Health Technology Summit—A Summary. *Telemedicine and e-Health*. 2011; 17(16): 501-506. Available at: https://doi.org/10.1089/tmj.2011.0088
Beyond Command and Control: USSOUTHCOM's use of Social Networking to 'Connect and Collaborate' During Haiti Relief Operations	Arias, R.	Arias, R. Beyond Command and Control: USSOUTHCOM's Use of Social Networking to 'Connect and Collaborate' During Haiti Relief Operations. In: Kumar, B. V. K. V., Prabhakar, S., Ross, A. A., Southern, S. O., Montgomery, K. N., Taylor, C. W., et al. (eds.) *Proceedings of SPIE Defence, Security, and Sensing, Volume 8029: Sensing Technologies for Global Health, Military Medicine, Disaster Response, and Environmental Monitoring; and Biometric Technology for Human Identification VIII; 25th-27th April 2011; Orlando, Florida, United States*. Washington: SPIE; 2011. Available at: https://doi.org/10.1117/12.884734
Beyond Smokestacks and Silos: Open-Source, Web-Enabled Coordination in Organizations and Networks	Roberts, N. C.	Roberts, N. C. Beyond Smokestacks and Silos: Open-Source, Web-Enabled Coordination in Organizations and Networks. *Public Administration Review*. 2011; 71(5): 677-693. Available at: https://doi.org/10.1111/j.1540-6210.2011.02406.x
Catastrophe and Containment: A Critical Analysis of the US Response to the 2010 Earthquake in Haiti	Moore, A.	Moore, A. Catastrophe and Containment: A Critical Analysis of the US Response to the 2010 Earthquake in Haiti. In: Attinà, F. (ed.) *The Politics and Policies of Relief, Aid and Reconstruction*. Basingstoke: Palgrave Macmillan; 2012. p.113-132. Available at: https://dx.doi.org/10.1057/9781137026736_7
Civil–Military Collaboration in the Initial Medical Response to the Earthquake in Haiti	Auerbach, P. S., Norris, R. L., Menon, A. S., Brown, I. P., Kuah, S., Schwieger, J., Kinyon, J., Helderman, T. N., and Lawry, L.	Auerbach, P. S., Norris, R. L., Menon, A. S., Brown, I. P., Kuah, S., Schwieger, J., et al. Civil–Military Collaboration in the Initial Medical Response to the Earthquake in Haiti. *New England Journal of Medicine*. 2010; 362(10): e32. Available at: https://dx.doi.org/10.1056/nejmp1001555
Civilian-Military Pooling of Health Care Resources in Haiti: A Theory of Complementarities Perspective	Naor, M., Dey, A., Meyer-Goldstein, S., and Rosen, Y.	Naor, M., Dey, A., Meyer-Goldstein, S., Rosen, Y. Civilian-Military Pooling of Health Care Resources in Haiti: A Theory of Complementarities Perspective. *International Journal of Production Research*. 2018; 56(21): 6741-6757. Available at: https://dx.doi.org/10.1080/00207543.2017.1355121
Collaboration in Humanitarian Logistics: Comparative Analysis of Disaster Response in Chile and Haiti 2010	Allende, V. and Anaya, J.	Allende, V., Anaya, J. *Collaboration in Humanitarian Logistics: Comparative Analysis of Disaster Response in Chile and Haiti 2010* [Master's Thesis]. California: Naval Postgraduate School; 2010. Available at: https://calhoun.nps.edu/handle/10945/10482
Collaborative Geospatial Data as Applied to Disaster Relief: Haiti 2010	Clark, A. J., Holliday, P., Chau, R., Eisenberg, H., and Chau, M.	Clark, A. J., Holliday, P., Chau, R., Eisenberg, H., Chau, M. Collaborative Geospatial Data as Applied to Disaster Relief: Haiti 2010. In: Kim, T.-h., Fang, W.-c., Khan, M. K., Arnett, K. P., Kang, H.-j., Slezak, D. (eds.) *Security Technology, Disaster Recovery and Business Continuity*. Communications in Computer and Information Science. Vol 122. Berlin, Germany: Springer; 2010. p.250-258. Available at: https://doi.org/10.1007/978-3-642-17610-4_29
Comparative Analysis of Emergency Response Operations: Haiti Earthquake in January 2010 and Pakistan's Flood in 2010	Niazi, J.I.K.	Niazi, J. I. K. *Comparative Analysis of Emergency Response Operations: Haiti Earthquake in January 2010 and Pakistan's Flood in 2010* [Master's Thesis]. California: Naval Postgraduate School; 2011. Available at: http://hdl.handle.net/10945/5516
Comparative Performance of Alternative Humanitarian Logistic Structures after the Port-au-Prince Earthquake: ACEs, PIEs, and CANs	Holguín-Veras, J., Jaller, M., and Wachtendorf, T.	Holguín-Veras, J., Jaller, M., Wachtendorf, T. Comparative Performance of Alternative Humanitarian Logistic Structures after the Port-Au-Prince Earthquake: ACEs, PIEs, and CANs. *Transportation Research Part A: Policy and Practice*. 2012; 46(10): 1623-1640. Available at: https://www.sciencedirect.com/science/article/pii/S0965856412001322
Coping with the Challenges of Early Disaster Response: 24 Years of Field Hospital Experience After Earthquakes	Bar-On, E., Abargel, A., Peleg, K., and Kreiss, Y.	Bar-On, E., Abargel, A., Peleg, K., Kreiss, Y. Coping with the Challenges of Early Disaster Response: 24 Years of Field Hospital Experience after Earthquakes. *Disaster Medicine and Public Health Preparedness*. 2013; 7(5): 491-498. Available at: https://dx.doi.org/10.1017/dmp.2013.94
Deployment of Field Hospitals to Disaster Regions: Insights from Ten Medical Relief Operations Spanning Three Decades	Naor, M., Heyman, S. N., Bader, T., and Merin, O.	Naor, M., Heyman, S. N., Bader, T., Merin, O. Deployment of Field Hospitals to Disaster Regions: Insights from Ten Medical Relief Operations Spanning Three Decades. *American Journal of Disaster Medicine*. 2017; 12(4): 243-256. Available at: https://doi.org/10.5055/ajdm.2017.0277
Dilemmas for Disaster Relief – The Cases of Myanmar, Haiti and Aceh through the Lens of National Sovereignty and International Intervention	Rucktäschel, K. and Schlegel, S.	Rucktäschel, K., Schlegel, S. Dilemmas for Disaster Relief—the Cases of Myanmar, Haiti and Aceh through the Lens of National Sovereignty and International Intervention. In: Neuhäuser, C., Schuck, C. (eds.) *Military Interventions: Considerations from Philosophy and Political Science*. 1st ed. Baden-Baden: Nomos Verlagsgesellschaft; 2017. p.107-128.
Disaster Aeromedical Evacuation	Lezama, N. G., Riddles, L. M., Pollan, W. A., and Profenna, L. C.	Lezama, N. G., Riddles, L. M., Pollan, W. A., Profenna, L. C. Disaster Aeromedical Evacuation. *Military Medicine*. 2011; 176(10): 1128-1132. Available at: https://dx.doi.org/10.7205/milmed-d-11-00040
Early Disaster Response in Haiti: The Israeli Field Hospital Experience	Kreiss, Y., Merin, O., Peleg, K., Levy, G., Vinker, S., Sagi, R., Abargel, A., Bartal, C., Lin, G., Bar, A., Bar-On, E., Schwaber, M.J., and Ash, N.	Kreiss, Y., Merin, O., Peleg, K., Levy, G., Vinker, S., Sagi, R., et al. Early Disaster Response in Haiti: The Israeli Field Hospital Experience. *Annals of Internal Medicine*. 2010; 153(1): 45-48. Available at: https://www.acpjournals.org/doi/abs/10.7326/0003-4819-153-1-201007060-00253
Emergency Knowledge Management and Social Media Technologies: A Case Study of the 2010 Haitian Earthquake	Yates, D. and Paquette, S.	Yates, D., Paquette, S. Emergency Knowledge Management and Social Media Technologies: A Case Study of the 2010 Haitian Earthquake. *International Journal of Information Management*. 2011; 31(1): 6-13. Available at: https://doi.org/10.1016/j.ijinfomgt.2010.10.001
Emerging Powers, Humanitarian Assistance and Foreign Policy: The Case of Brazil During the Earthquake Crisis in Haiti	Aguilar, S. L. C.	Aguilar, S. L. C. Emerging Powers, Humanitarian Assistance and Foreign Policy: The Case of Brazil During the Earthquake Crisis in Haiti. *International Journal of Humanities and Social Science*. 2012; 2(19): 93-101. Available at: http://hdl.handle.net/11449/115496
‘Going Back to History': Haiti and US Military Humanitarian Knowledge Production	Greenburg, J.	Greenburg, J. ‘Going Back to History': Haiti and US Military Humanitarian Knowledge Production. *Critical Military Studies*. 2018; 4(2): 121-139. Available at: https://dx.doi.org/10.1080/23337486.2017.1313380
Haiti Earthquake: Crisis and Response	Margesson, R. and Taft-Morales, M.	Margesson, R., Taft-Morales, M. *Haiti Earthquake: Crisis and Response* [Online] District of Columbia: Congressional Research Service; 2010. Available at: https://apps.dtic.mil/sti/citations/ADA516429
Haiti Relief: An International Effort Enabled through Air, Space, and Cyberspace	Fraser, D. M. and Hertzelle, W. S.	Fraser, D. M., Hertzelle, W. S. Haiti Relief: An International Effort Enabled through Air, Space, and Cyberspace. *Air & Space Power Journal*. 2010; 24(4): 5-12. Available at: https://apps.dtic.mil/sti/citations/ADA533555
Haiti: The US and Military Aid in Times of Natural Disaster (ARI)	Encina, C.G.	Encina, C. G. *Haiti: The US and Military Aid in Times of Natural Disaster (ARI)*. [Online] Madrid: Real Instituto Elcano; 2010. Available at: https://media.realinstitutoelcano.org/wp-content/uploads/2021/11/ari57-2010-garciaencina-haiti-us-militray-aid-natural-disaster.pdf
Haitian Earthquake Relief: Disaster Response Aboard the USNS Comfort	Walk, R. M., Donahue, T. F., Stockinger, Z., Knudson, M. M., Cubano, M., Sharpe, R. P., and Safford, S.D.	Walk, R. M., Donahue, T. F., Stockinger, Z., Knudson, M. M., Cubano, M., Sharpe, R. P., et al. Haitian Earthquake Relief: Disaster Response Aboard the USNS Comfort. *Disaster Medicine and Public Health Preparedness*. 2012; 6(4): 370-377. Available at: https://dx.doi.org/10.1001/dmp.2012.67
Healthcare Delivery Aboard Us Navy Hospital Ships Following Earthquake Disasters: Implications for Future Disaster Relief Missions	Sechriest II, V. F., Wing, V., Walker, G. J., Aubuchon, M., and Lhowe, D. W.	Sechriest II, V. F., Wing, V., Walker, G. J., Aubuchon, M., Lhowe, D. W. Healthcare Delivery Aboard US Navy Hospital Ships Following Earthquake Disasters: Implications for Future Disaster Relief Missions. *American Journal of Disaster Medicine*. 2012; 7(4): 281-294. Available at: https://doi.org/10.5055/ajdm.2012.0101
How Negotiations Within the Humanitarian Arena Shape the Effectiveness of the Coordination of Disaster Response: A Literature Review of the Indian Ocean Earthquake of 2004 in Indonesia and the Haitian Earthquake of 2010 in Haiti	Hoving, J. K.	Hoving, J. K. *How Negotiations within the Humanitarian Arena Shape the Effectiveness of the Coordination of Disaster Response: A Literature Review of the Indian Ocean Earthquake of 2004 in Indonesia and the Haitian Earthquake of 2010 in Haiti* [Master's Thesis]. Wageningen: Wageningen University; 2016. Available at: https://regroup-production.s3.amazonaws.com/documents/ReviewReference/213890334/edepotair_t58072658_001.pdf?response-content-type=application%2Fpdf&X-Amz-Algorithm=AWS4-HMAC-SHA256&X-Amz-Credential=AKIAYSFKCAWY23RWESRS%2F20220716%2Fus-east-1%2Fs3%2Faws4_request&X-Amz-Date=20220716T000639Z&X-Amz-Expires=604800&X-Amz-SignedHeaders=host&X-Amz-Signature=5dafeda35f1618009ba1343a799e2f511f4c1efc31c6e06362b66fa9926a47e9
Humanitarian Relief in Haiti, 2010: Honing the Partnership between the US Air Force and the UN	Owen, R. C.	Owen, R. C. Humanitarian Relief in Haiti, 2010: Honing the Partnership between the US Air Force and the UN. In: Dorn, A. W. (ed.) *Air Power in UN Operations: Wings for Peace*. 1st ed. Surrey: Ashgate Publishing; 2014. p.77-101. Available at: https://doi.org/10.4324/9781315566313
Independent Review of the U.S. Government Response to the Haiti Earthquake	Guha-Sapir, D., Kirsch, T., Dooling, S., Sirois, A., and DerSarkissian, M.	Guha-Sapir, D., Kirsch, T., Dooling, S., Sirois, A., DerSarkissian, M. *Independent Review of the U.S. Government Response to the Haiti Earthquake*. [Online] District of Columbia: United States Agency for International Development; 2011. Available at: https://pdf.usaid.gov/pdf_docs/pdacr222.pdf
Italy's Military Interventions and New Security Threats: The Cases of Somalia, Darfur and Haiti	Ceccorulli, M. and Coticchia, F.	Ceccorulli, M., Coticchia, F. Italy's Military Interventions and New Security Threats: The Cases of Somalia, Darfur and Haiti. *Contemporary Politics*. 2016; 22(4): 412-431. Available at: https://doi.org/10.1080/13569775.2016.1175095
Lessons from the Humanitarian Disaster Logistics Management: A Case Study of the Earthquake in Haiti	Salam, M. A. and Khan, S. A.	Salam, M. A., Khan, S. A. Lessons from the Humanitarian Disaster Logistics Management: A Case Study of the Earthquake in Haiti. *Benchmarking: An International Journal*. 2020; 27(4): 1455–1473. Available at: https://doi.org/10.1108/BIJ-04-2019-0165
Managing Airborne Relief During International Disasters	Morales, M. and Sandlin, D.E.	Morales, M., Sandlin, D. E. Managing Airborne Relief During International Disasters. *Journal of Humanitarian Logistics and Supply Chain Management*. 2015; 5(1): 12-34. Available at: https://doi.org/10.1108/JHLSCM-01-2014-0008
Military and Humanitarian Cooperation in Air Operations in Haiti	Whiting, M.C.	Whiting, M. C. Military and Humanitarian Cooperation in Air Operations in Haiti. *Humanitarian Exchange* [Online] 2012. February; 2012(53): 35-37. Available at: https://odihpn.org/wp-content/uploads/2012/03/humanitarianexchange053.pdf
Mobilizing for International Disaster Relief: Comparing U.S. and EU Approaches to the 2010 Haiti Earthquake	Brattberg, E. and Sundelius, B.	Brattberg, E., Sundelius, B. Mobilizing for International Disaster Relief: Comparing U.S. And EU Approaches to the 2010 Haiti Earthquake. *Journal of Homeland Security and Emergency Management*. 2011; 8(1): 0000102202154773551869. Available at: https://doi.org/10.2202/1547-7355.1869
Orthopedic Activity in Field Hospitals Following Earthquakes in Nepal and Haiti	Bar-On, E., Blumberg, N., Joshi, A., Gam, A., Peyser, A., Lee, E., Kashichawa, S.K., Morose, A., Schein, O., Lehavi, A., Kreiss, Y., and Bader, T.	Bar-On, E., Blumberg, N., Joshi, A., Gam, A., Peyser, A., Lee, E., et al. Orthopedic Activity in Field Hospitals Following Earthquakes in Nepal and Haiti. *World Journal of Surgery*. 2016; 40(9): 2117-2122. Available at: https://dx.doi.org/10.1007/s00268-016-3581-3
Partnered Disaster Preparedness: Lessons Learned From International Events	Born, C. T., Cullison, T. R., Dean, J. A., Hayda, R. A., McSwain, N., Riddles, L. M., and Shimkus, A. J.	Born, C. T., Cullison, T. R., Dean, J. A., Hayda, R. A., McSwain, N., Riddles, L. M., et al. Partnered Disaster Preparedness: Lessons Learned from International Events. *Journal of the American Academy of Orthopaedic Surgeons*. 2011; 19: S44-S48. Available at: https://journals.lww.com/jaaos/Fulltext/2011/02001/Partnered_Disaster_Preparedness__Lessons_Learned.10.aspx
Planning the Unplanned: The Role of a Forward Scout Team in Disaster Areas	Tarif, B., Merin, O., Dagan, D., and Yitzhak, A.	Tarif, B., Merin, O., Dagan, D., Yitzhak, A. Planning the Unplanned: The Role of a Forward Scout Team in Disaster Areas. *International Journal of Disaster Risk Reduction*. 2016; 19: 25-28. Available at: https://www.sciencedirect.com/science/article/pii/S2212420916302060
Relationships Matter: Humanitarian Assistance and Disaster Relief in Haiti	Keen, P. K., Neto, F. P. V., Nolan, C. W., Kimmey, J. L., and Althouse, J.	Keen, P. K., Neto, F. P. V., Nolan, C. W., Kimmey, J. L., Althouse, J. Relationships Matter: Humanitarian Assistance and Disaster Relief in Haiti. *Military Review*. 2010: 2-12. Available at: https://www.armyupress.army.mil/Portals/7/military-review/Archives/English/MilitaryReview_20100630_art004.pdf
Responding to Haiti	Dutton, G.	Dutton, G. Responding to Haiti. *World Trade*. 2010; 23(2): 16-17.
Response to the Humanitarian Crisis in Haiti Following the 12 January 2010 Earthquake: Achievements, Challenges and Lessons to Be Learned	Inter-Agency Standing Committee	Inter-Agency Standing Committee. *Response to the Humanitarian Crisis in Haiti Following the 12 January 2010 Earthquake: Achievements, Challenges and Lessons to Be Learned*. [Online] Geneva: Inter-Agency Standing Committee; 2010. Available at: https://reliefweb.int/report/haiti/response-humanitarian-crisis-haiti-following-12-january-2010-earthquake-achievements
Semantic and Social Networks Comparison for the Haiti Earthquake Relief Operations from APAN Data Sources Using Lexical Link Analysis (LLA)	Zhao, Y., Gallup, S.P., and MacKinnon, D.J.	Zhao, Y., Gallup, S. P., MacKinnon, D. J. Semantic and Social Networks Comparison for the Haiti Earthquake Relief Operations from APAN Data Sources Using Lexical Link Analysis (LLA). In: *Proceedings of the 17th International Command and Control Research and Technology Symposium (ICCRTS); 19th-21st June 2012; Fairfax, Virginia, United States*: ICCRTS; 2012. Available at: http://hdl.handle.net/10945/37505
Successes and Challenges of the Haiti Earthquake Response: The Experience of USAID	Weisenfeld, P. E.	Weisenfeld, P. E. Successes and Challenges of the Haiti Earthquake Response: The Experience of USAID. *Emory International Law Review*. 2011; 25(3): 1097-1120. Available at: https://scholarlycommons.law.emory.edu/eilr/vol25/iss3/3/
Telecommunications in Israeli Field Hospitals Deployed to Three Crisis Zones	Finestone, A. S., Levy, G., and Bar-Dayan, Y.	Finestone, A. S., Levy, G., Bar-Dayan, Y. Telecommunications in Israeli Field Hospitals Deployed to Three Crisis Zones. *Disasters*. 2014; 38(4): 833-845. Available at: https://dx.doi.org/10.1111/disa.12074
The Effects of Stabilisation on Humanitarian Action in Haiti	Muggah, R.	Muggah, R. The Effects of Stabilisation on Humanitarian Action in Haiti. *Disasters*. 2010; 34(S3): S444-S463. Available at: https://dx.doi.org/10.1111/j.1467-7717.2010.01205.x
The Haiti Earthquake Operation: Real Time Evaluation for the International Federation of Red Corss and Red Crescent Societies	Fisher, M., Bhattacharjee, A., Saenz, J., and Schimmelpfennig, S.	Fisher, M., Bhattacharjee, A., Saenz, J., Schimmelpfennig, S. *The Haiti Earthquake Operation: Real Time Evaluation for the International Federation of Red Corss and Red Crescent Societies*. [Online] Geneva: International Federation of Red Cross and Red Crescent Societies; 2010. Available at: https://www.ifrc.org/media/13753
The Islanding Effect: Post-Disaster Mobility Systems and Humanitarian Logistics in Haiti	Sheller, M.	Sheller, M. The Islanding Effect: Post-Disaster Mobility Systems and Humanitarian Logistics in Haiti. *Cultural Geographies*. 2013; 20(2): 185-204. Available at: https://dx.doi.org/10.1177/1474474012438828
The Use of Volunteer Interpreters During the 2010 Haiti Earthquake: Lessons Learned from the Usns Comfort Operation Unified Response Haiti	Powell, C. and Pagliara-Miller, C.	Powell, C., Pagliara-Miller, C. The Use of Volunteer Interpreters During the 2010 Haiti Earthquake: Lessons Learned from the Usns Comfort Operation Unified Response Haiti. *American Journal of Disaster Medicine*. 2012; 7(1): 37-47. Available at: https://doi.org/10.5055/ajdm.2012.0079
Tradeoffs Among Attributes of Resources in Humanitarian Operations: Evidence from United States Navy	Apte, A., Bacolod, M., and Carmichael, R.	Apte, A., Bacolod, M., Carmichael, R. Tradeoffs among Attributes of Resources in Humanitarian Operations: Evidence from United States Navy. *Production and Operations Management*. 2020; 29(4): 1071-1090. Available at: https://dx.doi.org/10.1111/poms.13154
Understanding Government Decision-Making: Canada's Disaster-Relief in Haiti and Pakistan	Mamuji, A. A.	Mamuji, A. A. *Understanding Government Decision-Making: Canada's Disaster-Relief in Haiti and Pakistan* [Doctoral Dissertation]. Ottawa: University of Ottawa; 2014. Available at: https://ruor.uottawa.ca/bitstream/10393/31704/5/Mamuji_Aaida_2014_thesis.pdf
United Nations–European Union Cooperation in Aid, Relief and Reconstruction — The Haiti Case	Morsut, C. and Iturre, M. J.	Morsut, C., Iturre, M. J. United Nations–European Union Cooperation in Aid, Relief and Reconstruction — the Haiti Case. In: Attinà, F. (ed.) *The Politics and Policies of Relief, Aid and Reconstruction*. London: Palgrave Macmillan; 2012. p.133-150. Available at: https://dx.doi.org/10.1057/9781137026736_8
Using Web 2.0 Technology to Support Humanitarian Assistance and Disaster Relief Operations: Applying the Lessons Learnt from the United States Military Response to the 2010 Haiti Earthquake to Improve the Utilisation of the New Zealand Defence Force's Communications and Information Systems During Humanitarian Assistance and Disaster Relief Operations	Jones, L. S.	Jones, L. S. *Using Web 2.0 Technology to Support Humanitarian Assistance and Disaster Relief Operations: Applying the Lessons Learnt from the United States Military Response to the 2010 Haiti Earthquake to Improve the Utilisation of the New Zealand Defence Force's Communications and Information Systems During Humanitarian Assistance and Disaster Relief Operations* [Master's Thesis]. Manawatu: Massey University; 2011. Available at: https://mro.massey.ac.nz/bitstream/handle/10179/4265/02_whole.pdf

Listed alphabetically, by article title.

**Table 3:** Grey literature: Articles included.

**Title**	**Author(s)**	**Source**
22d MEU Unified Response CONOP Brief	22nd Marine Expeditionary Unit	**Grey literature:** 22nd Marine Expeditionary Unit. *22d MEU Unified Response CONOP Brief*. [Presentation] United States Southern Command. 19th January 2010.
Action Memorandum: Operation Unified Response Quick-Look Assessment Report	Haley, J.R.	**Grey literature:** Haley, J. R. *Action Memorandum: Operation Unified Response Quick-Look Assessment Report*. 15th March 2010.
After Action Review - Operation Unified Response, Lessons Learned: SCJ4 Operational Contract Support	United States Southern Command	**Grey literature:** United States Southern Command. *After Action Review - Operation Unified Response, Lessons Learned: SCJ4 Operational Contract Support*. Florida: United States Southern Command: 2010.
Building Habitability Assessment Plan	Joint Task Force-Haiti	**Grey literature:** Joint Task Force-Haiti. *Building Habitability Assessment Plan*. [Presentation] 2010.
Commander United States Southern Command Executive Order, 18 January 2010	Commander United States Southern Command	**Grey literature:** Commander United States Southern Command. *Commander United States Southern Command Executive Order, 18 January 2010*. Florida: United States Southern Command; 18th January 2010.
Commander United States Southern Command For Official Use Only, Order 16 January 2010	Commander United States Southern Command	**Grey literature:** Commander United States Southern Command. *Commander United States Southern Command for Official Use Only, Order 16 January 2010*. Florida: United States Southern Command; 16th January 2010.
Consolidated Southern Command Fragmentary Orders: Lessons Learned	Commander 4th Fleet	**Grey literature:** Commander 4th Fleet. *Consolidated Southern Command Fragmentary Orders: Lessons Learned*. Florida: United States Southern Command; 2010. Report Number: 091.
Department of Defense Support to Foreign Disaster Relief: Handbook for JTF Commanders and Below	United States Department of Defense	**Grey literature:** United States Department of Defense. *Department of Defense Support to Foreign Disaster Relief: Handbook for JTF Commanders and Below*. District of Columbia: United States Government Printing Office; 2011.
Draft: Operation Unified Response (OUR) AAR	United States Southern Command	**Grey literature:** United States Southern Command. *Draft: Opeartion Unified Response (OUR) AAR*. [Presentation] United States Southern Command. 2010.
Emergency Response after the Haiti Earthquake: Choices, Obstacles and Finance	Médecins Sans Frontières	**Grey literature:** Médecins Sans Frontières. *Emergency Response after the Haiti Earthquake: Choices, Obstacles and Finance*. [Online] Geneva: Médecins Sans Frontières; 2010. Available at: https://www.msf.org/emergency-response-after-haiti-earthquake-choices-obstacles-and-finance
Haiti after the Disaster – Lessons learned from Evaluations, Consequences and Recommendations for the Future of Swiss Humanitarian Aid	Tobler, C., Hasler, N., and Chastonay, C.	**Grey literature:** Tobler, C., Hasler, N., Chastonay, C. *Haiti after the Disaster – Lessons Learned from Evaluations, Consequences and Recommendations for the Future of Swiss Humanitarian Aid* [Unpublished Coursework]. St. Gallen: University of St. Gallen; 2011.
Haiti Earthquake After Action Report and Lessons Learned (AAR/LL): Hastily Formed Networks in Haiti	Steckler, B.	**Grey literature:** Steckler, B. *Haiti Earthquake After Action Report and Lessons Learned (AAR/LL): Hastily Formed Networks in Haiti*. California: Naval Postgraduate School, Hastily Formed Networks Center; 8th September 2010. Available at: https://nps.edu/documents/105738171/0/Haiti+Earthquake+AAR-LL+Document+-+Steckler+NPS+HFN+Center+-+10+SEP+2010.pdf/caceb389-e228-495d-bc89-d8d7cfd79f64
Haiti Earthquake Relief: One-Year Report	American Red Cross	**Grey literature:** American Red Cross. *Haiti Earthquake Relief: One-Year Report*. [Online] District of Columbia: American Red Cross; 2011. Available at: https://www.redcross.org/content/dam/redcross/atg/PDF_s/HaitiEarthquake_OneYearReport.pdf
Haiti Lessons Learned: Operation Unified Response	Branch, T.	**Grey literature:** Branch, T. *Haiti Lessons Learned: Operation Unified Response*. [Presentation] Carrier Strike Group 1. 8th April 2010.
Haiti: Carrier Strike Group-1 Operations Order	Commander Carrier Strike Group-1	**Grey literature:** Commander Carrier Strike Group-1. *Haiti: Carrier Strike Group-1 Operations Order*. California: United States 3rd Fleet; 15th January 2010. Report Number: 100116.
Health Response to the Earthquake in Haiti: January 2010	Goyet, C. d. V. d., Sarmiento, J. P., and Grünewald, F.	**Grey literature:** Goyet, C. d. V. d., Sarmiento, J. P., Grünewald, F. *Health Response to the Earthquake in Haiti: January 2010*. [Online] Washington: Pan American Health Organization; 2011. Available at: https://iris.paho.org/bitstream/handle/10665.2/52841/9789275132524_eng.pdf?sequence=1&isAllowed=y
HQ USSOUTHCOM: Operation Unified Response AAR	United States Southern Command	**Grey literature:** United States Southern Command. *HQ USSOUTHCOM: Operation Unified Response AAR*. [Presentation] United States Southern Command. 10th May 2010.
JTF-Haiti Recommendation to Release USNS Comfort	United States Southern Command	**Grey literature:** United States Southern Command. *JTF-Haiti Recommendation to Release USNS Comfort* [Presentation] United States Southern Command. 25th February 2010.
JTF-Haiti: CVN Departure Assessment	United States Southern Command	**Grey literature:** United States Southern Command. *JTF-Haiti: CVN Departure Assessment*. [Presentation] United States Southern Command. 26th January 2010.
Meeting Minutes: Joint Chiefs of Staff Brief 19th January 2010	Joint Chiefs of Staff	**Grey literature:** Joint Chiefs of Staff. *Meeting Minutes: Joint Chiefs of Staff Brief 19th January 2010*. District of Columbia: United States Department of Defense; 19th January 2010.
Memorandum for Heads of Executive Departments and Agencies: Special Solicitation for Haitian Earthquake Relief	Berry, J.	**Grey literature:** Berry, J. *Memorandum for Heads of Executive Departments and Agencies: Special Solicitation for Haitian Earthquake Relief*. District of Columbia: United States Office of Personnel Management; 14th January 2010.
Minutes of the Meeting of Joint Task Force-Haiti Commander's Conference	Commander 4th Fleet	**Grey literature:** Commander 4th Fleet. *Minutes of the Meeting of Joint Task Force-Haiti Commander's Conference*. Florida: United States Naval Forces Southern Command; 13th February 2010.
Modification 1 to United States Southern Command Executive Order: Operation Unified Response	Fraser, D.	**Grey literature:** Fraser, D. *Modification 1 to United States Southern Command Executive Order: Operation Unified Response*. Florida: United States Southern Command; 17th January 2010. Report Number: MSG/CDRUSSOUTHCOM/161330ZJAN10.
Modification 4 to United States Southern Command Executive Order: Operation Unified Response	Fraser, D.	**Grey literature:** Fraser, D. *Modification 4 to United States Southern Command Executive Order: Operation Unified Response*. Florida: United States Southern Command; 19th January 20110. Report Number: MSG/CDRUSSOUTHCOM/190032ZJAN10.
Operation Haiti Relief: After Action Report	Florida State Emergency Response Team	**Grey literature:** Florida State Emergency Response Team. *Operation Haiti Relief: After Action Report*. Florida: Florida Division of Emergency Management; 2010.
Operation Unified Response – Haiti Earthquake 2010	DiOrio, D.R.	**Grey literature:** DiOrio, D. R. *Operation Unified Response – Haiti Earthquake 2010*. [Online] Virginia: Joint Forces Staff College; 2010. Available at: https://jfsc.ndu.edu/Portals/72/Documents/JC2IOS/Additional_Reading/4A_Haiti_HADR_Case_Study_revNov10.pdf
Operation Unified Response (Haiti Earthquake): After Action Report	United States Coast Guard: Atlantic Area	**Grey literature:** United States Coast Guard: Atlantic Area. *Operation Unified Response (Haiti Earthquake): After Action Report*. Virginia: United States Coast Guard; 2011.
Operation Unified Response (Haiti Earthquake): After Action Review	7th Sustainment Brigade	**Grey literature:** 7th Sustainment Brigade. *Operation Unified Response (Haiti Earthquake): After Action Review*. 23rd June 2010.
Operation Unified Response (Haiti): CDR's Update Brief	Commander United States Naval Forves Southern Command	**Grey literature:** Commander United States Naval Forves Southern Command. *Operation Unified Response (Haiti): CDR's Update Brief*. [Presentation] United States Naval Forces Southern Command. 20th Jan 2010.
Operation Unified Response (OUR): Compendium of USAF Reports	Henningsen, J. R. (Editor)	**Grey literature:** Henningsen, J. R. (ed.) *Operation Unified Response (OUR): Compendium of USAF Reports*. District of Columbia: United States Air Force, Studies and Analyses, Assessments and Lessons Learned; 2011.
Operation Unified Response: A Case Study of the Military's Role in Disaster Relief Operations	Hughes, T. D.	**Grey literature:** Hughes, T. D. *Operation Unified Response: A Case Study of the Military's Role in Disaster Relief Operations* [Master's Thesis]. Virginia: Marine Corps University; 2011. Available at: https://apps.dtic.mil/sti/pdfs/ADA600734.pdf
Operation Unified Response: Air Mobility Command's Response to the 2010 Haiti Earthquake Crisis	Wallwork, E. D., Gunn, K. S., Morgan, M. L., and Wilcoxson, K. A.	**Grey literature:** Wallwork, E. D., Gunn, K. S., Morgan, M. L., Wilcoxson, K. A. *Operation Unified Response: Air Mobility Command's Response to the 2010 Haiti Earthquake Crisis*. [Online] Illinois: Office of History, Air Mobility Command; 2010. Available at: https://www.amc.af.mil/Portals/12/documents/AFD-131018-050.pdf
Operation Unified Response: Haiti Earthquake Response	Joint Center for Operational Analysis	**Grey literature:** Joint Center for Operational Analysis. *Operation Unified Response: Haiti Earthquake Response*. [Presentation] Joint Center for Operational Analysis. May 2010.
Operation Unified Response: Haiti Earthquake Situation Update	United States Department of Defense	**Grey literature:** United States Department of Defense. *Operation Unified Response: Haiti Earthquake Situation Update*. [Presentation] District of Columbia: United States Department of Defense. 19th January 2010.
Operation Unified Response: Humanitarian Assistance Response Force (HARF)	Commander United States Southern Command	**Grey literature:** Commander United States Southern Command. *Operation Unified Response: Humanitarian Assistance Response Force (HARF)*. [Presentation] United States Southern Command. 19th February 2010.
Operation Unified Response: Joint Task Force Port Opening/Commander Task Force 42	United States Southern Command	**Grey literature:** United States Southern Command. *Operation Unified Response: Joint Task Force Port Opening/Commander Task Force 42*. [Presentation] United States Southern Command. 12th February 2010.
Operation Unified Response: JTF-H Concept Brief	Campbell, J.	**Grey literature:** Campbell, J. *Operation Unified Response: JTF-H Concept Brief*. [Presentation] United States Southern Command. 22nd January 2010.
Operation Unified Response: Transition Strategy	United States Southern Command	**Grey literature:** United States Southern Command. *Operation Unified Response: Transition Strategy*. [Presentation] United States Southern Command. 12th February 2010.
Operation Unified Response: Transition to Long Term Engagement	Alvirez, S.	**Grey literature:** Alvirez, S. *Operation Unified Response: Transition to Long Term Engagement*. [Presentation] United States Southern Command. 2010.
Proceedings for Operation Unified Response – Haiti Navy Medicine After Action Review	Valentin, E. V. (Editor)	**Grey literature:** Valentin, E. V. (ed.) Proceedings for Operation Unified Response – Haiti Navy Medicine After Action Review. *Operation Unified Response – Haiti Navy Medicine After Action Review; 5th-6th May 2010*; Maryland, United States. Texas: Navy Medicine Support Command; 2010.
Public Health Risk Assessment and Interventions - Earthquake: Haiti	World Health Organisation	**Grey literature:** World Health Organisation. *Public Health Risk Assessment and Interventions - Earthquake: Haiti*. [Online] Geneva: World Health Organisation: Disease Control in Humanitarian Emergencies; 2010. Available at: https://reliefweb.int/report/haiti/public-health-risk-assessment-and-interventions-earthquake-haiti-21-january-2010
Some Challenges and Considerations in Forming a Joint Task Force	Joint Center for Operational Analysis	**Grey literature:** Joint Center for Operational Analysis. *USSOUTHCOM and JTF-Haiti: Some Challenges and Considerations in Forming a Joint Task Force*. Virginia: United States Joint Forces Command; 2010.
Stability Operations in Haiti 2010: A Case Study	Vialpando, E.	**Grey literature:** Vialpando, E. *Stability Operations in Haiti 2010: A Case Study*. [Online] Pennsylvania: Peacekeeping and Stability Operations Institute; 2016. Available at: https://publications.armywarcollege.edu/pubs/3306.pdf
The U.S. Military Response to the 2010 Haiti Earthquake: Considerations for Army Leaders	Cecchine, G., Morgan, F. E., Wermuth, M. A., Jackson, T., Schaefer, A. G., and Stafford, M.	**Grey literature:** Cecchine, G., Morgan, F. E., Wermuth, M. A., Jackson, T., Schaefer, A. G., Stafford, M. *The U.S. Military Response to the 2010 Haiti Earthquake: Considerations for Army Leaders*. [Online] California: RAND Corporation; 2013. Available at: https://www.rand.org/pubs/research_reports/RR304.html
USAID Haiti Earthquake Taskforce: (SBU) Situation Report No. 11	United States Agency for International Development	**Grey literature:** United States Agency for International Development. *USAID Haiti Earthquake Taskforce: (SBU) Situation Report No. 11*. District of Columbia: United States Agency for International Development; 18th January 2010. Report Number: 11.
USAID Knowledge Services Center (KSC): Lessons Learned from the 2005 Pakistan Earthquake	United States Agency for International Development	**Grey literature:** United States Agency for International Development. *USAID Knowledge Services Center (KSC): Lessons Learned from the 2005 Pakistan Earthquake*. [Online] District of Columbia: United States Agency for International Development: Knowledge Services Center; 2010. Available at: https://pdf.usaid.gov/pdf_docs/PNADM100.pdf

Listed alphabetically, by article title.

**Table 4:** Haitian literature search: Articles included.

**Title**	**Author(s)**	**Source**
Anaesthetic Safety, from Humanitarian to Development	Fabien, D.	**Haitian literature:** Fabien, D. Anaesthetic Safety, from Humanitarian to Development. *INFO-CHIR: La Revue Haitienne de Chirurgie et d'Anesthésiologie*. 2012; 2(8): 19-21.
Culturally Competent Volunteer Becomes a Partner after the Earthquake	Tascoe. R. M.	**Haitian literature:** Tascoe. R. M. Culturally Competent Volunteer Becomes a Partner after the Earthquake. *INFO-CHIR: La Revue Haitienne de Chirurgie et d'Anesthésiologie*. 2011; 1 (4): 29-33.
Genitourinary Trauma in Disaster Situations: The Haitian Earthquake of January 12, 2010	Gousse, A. E.	**Haitian literature:** Gousse, A. E. Genitourinary Trauma in Disaster Situations: The Haitian Earthquake of January 12, 2010. *INFO-CHIR: La Revue Haitienne de Chirurgie et d'Anesthésiologie*. 2011; 1(4): 4-7.

Listed alphabetically, by article title.

**Table 5:** Citation searches: Articles included.

**Title**	**Author(s)**	**Source**
Foreign Disaster Response: Joint Task Force–Haiti Observations	Keen, P. K., Elledge, M. G., Nolan, C. W., and Kimmey, J. L.	**Citation search:** Keen, P. K., Elledge, M. G., Nolan, C. W., Kimmey, J. L. Foreign Disaster Response: Joint Task Force–Haiti Observations. *Military Review*. 2010; November-December: 85-96. Available at: https://apps.dtic.mil/sti/citations/ADA537030
Haiti Earthquake 2010: One-Year Progress Report	International Federation of Red Cross And Red Crescent Societies	**Citation search:** International Federation of Red Cross And Red Crescent Societies. *Haiti Earthquake 2010: One-Year Progress Report*. [Online] Geneva: International Federation of Red Cross And Red Crescent Societies; 2011. Available at: https://reliefweb.int/report/haiti/haiti-earthquake-2010-one-year-progress-report
The Logistic Experience of the Brazilian Navy in Humanitarian Operations: The Cases of Earthquakes in Haiti and Chile in 2010	Mendonça, B. G. S. G. d., Paula-Filho, A. B. d., and Leiras, A.	**Citation search:** Mendonça, B. G. S. G. d., Paula-Filho, A. B. d., Leiras, A. The Logistic Experience of the Brazilian Navy in Humanitarian Operations: The Cases of Earthquakes in Haiti and Chile in 2010. *Production*. 2019; 29: e20170082. Available at: https://dx.doi.org/10.1590/0103-6513.20170061
The United Nations Humanitarian Civil–Military Coordination (UN–CMCoord) Response to the Haiti Earthquake	Butterfield, A., Reario, R., and Dolan, R.	**Citation search:** Butterfield, A., Reario, R., Dolan, R. *The United Nations Humanitarian Civil–Military Coordination (UN–CMCoord) Response to the Haiti Earthquake*. Humanitarian Exchange [Online] 2010. October; 2010(48): 13-15. Available at: https://odihpn.org/wp-content/uploads/2010/08/humanitarianexchange048.pdf

Listed alphabetically, by article title.

## Results

### The humanitarian and military response

#### International dominance

The international response to the 2010 earthquake, constituted the largest humanitarian intervention carried out within a single nation (16). More than 140 governments, and over 1,000 NGOs, offered assistance (2, 9). A total of 26 nations sent military forces, the largest military cadre being that of the US (19)—who initially deployed 13,000 troops (20), a number that reached 22,000 during peak phases of the responses (2, 9, 19, 21).

The literature universally highlights the “International Nature” of the humanitarian response. Discussion encompasses international governments and the UN (16, 20, 22–25), international NGOs (2, 6, 19, 25–27), and international military organisations (9, 20, 24, 28–32)—predominantly, the activities of the US military (1, 2, 9, 13, 16, 19–21, 26, 31–59). What starkly manifests in the literature, is the paucity of discussion of the Haitian contribution to the response. There was limited inclusion of Haitian achievements—which, when discussed, consisted mainly of statements that work had been conducted alongside the Government of Haiti (GoH) (60), agreements and strategy had been formed with assistance from the GoH (36), or that support was to be provided to the GoH (24, 38, 44, 47). This is surprising, given that over 800 civil society organisations existed in Haiti, prior to the disaster (6).

#### The medical response

The 2010 earthquake resulted in over 316,000 deaths, and 300,000 injured casualties (12). This inordinate burden of traumatically injured patients, initially overwhelmed local facilities (29). Therefore, a core aspect of the humanitarian response was to facilitate delivery of emergency medical care to the victims. The enormity of the medical efforts undertaken during this response, cannot be overstated. Twenty-four days after the earthquake occurred, 91 hospitals, including 21 Foreign Field Hospitals (FFH), and five hospital ships, were operational within Haiti (14) (Tables 6–8).

**Table 6:** Summary of healthcare operations: United States military.

	**Operation Unified Response**	**AFSOC**	**USS Bataan (22nd MEU)**	**USS** **Carl Vinson**	**USNS Comfort**	**SPEARR**	**EMEDS (6th air mobility wing)**	**USS Nassau** **(24th MEU)**
Date of Arrival	January 13th	January 13th	January 18th	January 15th	January 20th	Initial Team: January 23rd Replacement Team: Mid-March	January 24th	January 23rd
Date of Departure	Officially Concluded June 1st 2010	January 23rd	March 25th	February 1st	March 10th	June 1st	March 19th	February 9th
Capacity	Ships Deployed: 33 Aircraft Deployed: 130	–	Overflow Beds: 540 Ward Beds: 47 ICU Beds: 17 Operating theatres: 6	Total Beds: 50 ICU Beds: 8–9	Total Beds: 1,000 CASREC Beds: 50 ICU Beds: 60–80 Recovery Beds: 20 Operating Theatres: 12–20	Critical Care Beds: 10 ICU Beds: 3	Ward Beds: 20 Critical Care Beds: 3 Operating Theatres: 1	–
Staffing	Max Personnel: 22,000	–	GS: 3 T&O: 2 O&G: 1 O/MF:1 AN: 3 SN: 1	–	Total Medical Personnel: ≈ 400 Interpreters: 130 (57 Navy, 73 ARC)	Total: 12 T&O: 1 GS: 1 AN: 1 EM: 1 PH: 1 IM: 1 AeSp: 1 SN: 1 CCN: 1 CPT: 1 BMS: 1 PHT:1	Total: 78 Surgical Team: 5 T&O: 1 GS: 1 AN: 1 EM: 1 SN: 1	-
Patients Triaged	–	8,000	–	–		–	–	–
Patients Treated	19,000	362	47	–	Total: 872 Outpatient: 55	–	2,500	>100
Surgical Operations	Procedures: 1,025	Procedures: 14	Procedures: 109	–	Procedures: 927 - Patients: 454 - Extremity Injuries: 669 - Craniofacial • Reconstruction: 93	Procedures: 10 - Not performed at airport site - Surgeons volunteered at local NGO units	Procedures: 12	–
Primary Specialties by % of Operative Cases	–	–	T&O: 55% GS: 29%	–	T&O: 55% GS: 9%	–	–	–
Amputations	–	9	4	–	Primary Amputations: 37 Revision Surgeries: 105 (58 Patients)	–	–	–
Inpatient Admissions	–	–	–	–	Total: 817 Haitian Nationals: 773 US Military: 26 US Civilians: 15 Canadian Military: 3	–	150	–
Patient Transfers	2,200	–	–	–	Transferred to Haitian Facilities for Continued Care: 448	–	500	–
Evacuations	Medical Evacuations: 343 US Citizen Evacuations: 16,412	Total: 167	Total: 500	–	Total Evacuated to US: 77 Haitians Evacuated to US: 69	Total: 498	–	–

“–”, Information Unavailable; ICU, Intensive Care Unit; GS, General Surgery/Surgeon; T&O, Trauma and Orthopaedics/Trauma and Orthopaedic Surgeon; O&G, Obstetrician and Gynaecologist; O/MF, Oral and Maxillofacial Surgery/Surgeon; AN, Anaesthetics/Anaesthetist; EM, Emergency Physician(s); PH, Public Health Specialist; IM, Internal Medicine Physician(s); M&D, Medical and Dental; AeSp, Aerospace Medical Specialist(s); SN, Scrub Nurse(s)/Theatre Nurse(s); CCN, Critical Care Nurse(s); CPT, Cardiopulmonary Technician(s); BMS, Biomedical Scientist(s); PHT, Public Health Technician(s); ARC, American Red Cross; DRC, Dominican Red Cross; NRC, Norwegian Red Cross.

**Table 7:** Summary of healthcare operations: international military organisations—non-US.

	**Spanish Armed Forces (Castilla)**	**French Armed Forces (Siroco)**	**Military Forces of Colombia (Cartagena de Indias)**	**Mexican Armed Forces** ** (Huasteco)**	**Canadian Armed Forces (HMCS Athabaskan)**	**Brazilian Air Force Foreign Field Hospital**	**Israel Defense Forces Foreign Field Hospital**	**Canadian Armed Forces Foreign Field Hospital**
Date of arrival	February 4th	January 24th	January 22nd	January 20th	January 19th	–	January 13th	January 29th
Date of Departure	May 4th	February 6th	February 14th	–	–	–		
Capacity	Total Beds: 70 Ward Beds: 62 ICU Beds: 8 Operating Theatres: 2	Total Beds: 50 Operating Theatres: 2	Operating Theatres: 1	Total Beds: 25 Operating Theatres: 1	–		–	Ward Beds: 100 ICU Beds: 4 Operating Theatres: 2
Staffing		–	Physicians: 8	–	Total: 250–300	Physicians: 26	Total: 121 Physicians: 44	M&D Personnel: 97 Surgical Teams: 2 T&O: 1 GS: 1 AN: 1 SN: 1
Patients Triaged	–	–	–	–	–	–	–	–
Patients Treated	7,568	–	200	–	–	36,028	1,111 Fractures: 265	4,922
Inpatient Admissions	–	–			–	–	737	–
Surgical Operations	Procedures: 104	Procedures: 45	Procedures: 27	–	–	1,145	Procedures: 244	Procedures: 167 Inguinal Hernia and Hydrocoele Repairs: 69 Internal Fixation: 12 External Fixation: 7
Primary Specialties by % of Operative Cases	–	–	–	–	–	–	T&O: 83%	–
Amputations	–	–	–	–	–	–	–	6
Patient Transfers	–	–	–	–	–	–	–	-
Evacuations	–	–	–	–	–	–	–	–

“–”, Information Unavailable; ICU, Intensive Care Unit; GS, General Surgery/Surgeon; T&O, Trauma and Orthopaedics/Trauma and Orthopaedic Surgeon; O&G, Obstetrician and Gynaecologist; O/MF, Oral and Maxillofacial Surgery/Surgeon; AN, Anaesthetics/Anaesthetist; EM, Emergency Physician(s); PH, Public Health Specialist; IM, Internal Medicine Physician(s); M&D, Medical and Dental; AeSp, Aerospace Medical Specialist(s); SN, Scrub Nurse(s)/Theatre Nurse(s); CCN, Critical Care Nurse(s); CPT, Cardiopulmonary Technician(s); BMS, Biomedical Scientist(s); PHT, Public Health Technician(s); ARC, American Red Cross; DRC, Dominican Red Cross; NRC, Norwegian Red Cross.

**Table 8:** Summary of healthcare operations: international organisations—civilian.

	**Médecins Sans Frontières**	**The International Federation of Red Cross and Red Crescent Societies**	**Dominican Republic Emergency Teams**	**University of Miami** ** Project Medishare Hospital**	**Cuban Medical Brigade**
Date of arrival	January 13th	January 12th (DRC)	January 12th	January 21st	January 12th
Date of departure	–	–	–	–	–
Capacity	Hospitals: 2 Fixed Sites: 19 Mobile Units: 3 Total Beds: 1,187 Operating Theatres: 16	FFH: 2 FFH (NRC) - Total Beds: 20 Basic Health Care Units: 4 Fixed Sites: 4 Mobile Units: 41	–	250	–
Staffing	Haitian Staff: 2,807 International Staff: 209	FFH (NRC) Personnel: 30 Surgical Teams: 2 Outpatient Teams: 1 Haitian Volunteers Trained: 20 Mental Health, 110 Vaccinators	–	Total: 12	Total: 1,500
Patients triaged	–	–	–	–	Within 1st 24 h: 1,000 Total: 20,095
Patients treated	173,757	216,900	2,000	–	14,551
Inpatient admissions	–	–	–		
Surgical operations	11,748	1,339 FFH (NRC): 300	–	–	1,252
Primary specialties by % of operative cases	–	–	–	–	–
Amputations	Within 1st 20 Days: 140	–	–	–	–
Patient transfers	–	–	–	–	–
Evacuations	–	–	–	–	–

“–”, Information Unavailable; ICU, Intensive Care Unit; GS, General Surgery/Surgeon; T&O, Trauma and Orthopaedics/Trauma and Orthopaedic Surgeon; O&G, Obstetrician and Gynaecologist; O/MF, Oral and Maxillofacial Surgery/Surgeon; AN, Anaesthetics/Anaesthetist; EM, Emergency Physician(s); PH, Public Health Specialist; IM, Internal Medicine Physician(s); M&D, Medical and Dental; AeSp, Aerospace Medical Specialist(s); SN, Scrub Nurse(s)/Theatre Nurse(s); CCN, Critical Care Nurse(s); CPT, Cardiopulmonary Technician(s); BMS, Biomedical Scientist(s); PHT, Public Health Technician(s); ARC, American Red Cross; DRC, Dominican Red Cross; NRC, Norwegian Red Cross.

##### Military-humanitarian response

In total, 26 nations contributed military personnel, the largest of which was the US (19)—whose joint effort was termed, Operation Unified Response (OUR). During OUR, the joint components of the US military delivered health care to around 19,000 victims, performed 1,025 operations, and provided 70,000 medical prescriptions (9). They also participated in 2,200 patient transfers and distributed around 75 tonnes of medical equipment (9).

**Figure 4:**
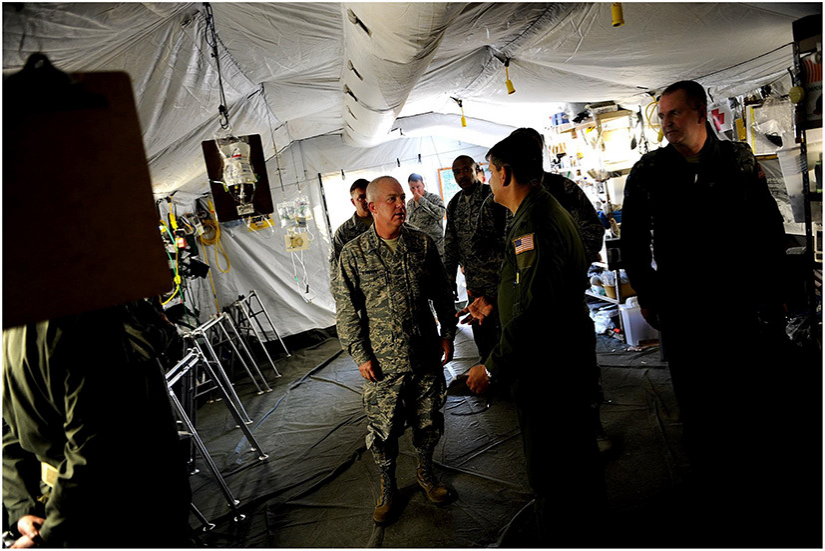
Inside the AFSOC medical tent, U.S. Air Force AFSOC Commander Lt. Gen. Donald C. Wurster visits with his troops at the Toussaint Louverture International Airport, Port-au-Prince, Haiti, on January 27 during Operation Unified Response. DoD assets have been deployed to assist in the Haiti relief effort following a magnitude 7 earthquake that hit the city on January 12. The appearance of U.S. DoD visual information does not imply or constitute DoD endorsement. Source: Public domain image, not in copyright. Available at: https://commons.wikimedia.org/wiki/File:Operation_Unified_Response_DVIDS244961.jpg.

The US Air Force (USAF) provided initial medical response and evacuation capabilities (33) within 24 h of the disaster (40). The initial response unit consisted of an Air Force Special Operation Command (AFSOC) team—supported by surgical, critical care, and medical assets (40). Of the AFSOC teams deployed, one remained at the airport with the critical care and evacuation team (Figure 4), whilst the other responded to the American embassy (40). The embassy team triaged over 8,000 American citizens, treated 362 patients, and performed 14 major operations, 9 of which were amputations (40). The Small Portable Expeditionary Aeromedical Rapid Response (SPEARR) team, arrived on January 23rd and replaced the initial AFSOC team at *Port-au-Prince-Toussaint L'Ouverture International Airport* (MTPP) (40). The SPEARR team consisted of twelve members, who evacuated 498 patients over their 2-month deployment (40). The final USAF asset deployed, was the 78-member team, of the Expeditionary Medical Support (EMEDS) system (40). EMEDS personnel arrived on January 24th, primarily setting up at a private seaport, Terminal Varreux (40). Their team treated over 2,500 patients-−150 of which required inpatient admission—participated in over 500 patient transfers, and conducted 12 operative procedures (40).

**Figure 5:**
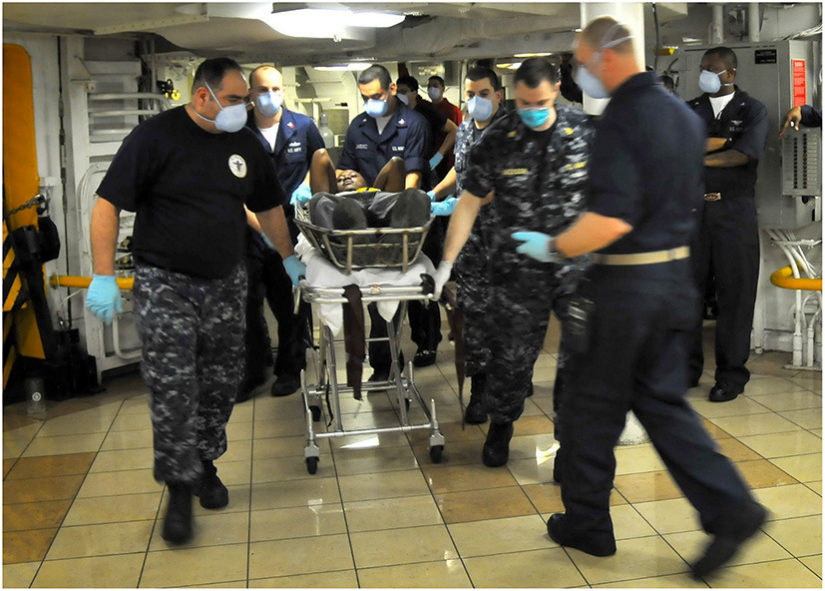
A medical response team aboard the Nimitz-class aircraft carrier USS Carl Vinson (CVN 70) transports a Haitian patient to an operating room after being flown aboard by helicopter. Carl Vinson and Carrier Air Wing 17 are conducting humanitarian and disaster relief operations as part of Operation Unified Response after a 7.0 magnitude earthquake caused severe damage near Port-au-Prince, Haiti, January 12, 2010 (U.S. Navy photo by Mass Communication Specialist 2nd Class Daniel Barker/Released). The appearance of U.S. DoD visual information does not imply or constitute DoD endorsement. Source: Public domain image, not in copyright. Available at: https://commons.wikimedia.org/wiki/File:USS_Carl_Vinson_relief_operations_100112-N-RI884-065.jpg.

Within 4 days of the earthquake, the US Navy (USN) was able to begin treating patients on the USS Carl Vinson (47) (Figure 5). Following this, the largest sea-based asset involved in the disaster response, the hospital ship USNS Comfort (29), arrived January 20th, with tertiary care capability. The USNS Comfort's capabilities included at least 30 medical sub-specialties, supplemented by physiotherapists, nurse practitioners, midwives and physician's assistants—totalling almost 400 medical staff (39). Over 90% of the US military's surgical procedures were carried out onboard, the vast majority of which, were for extremity injuries (39). Of the injuries that presented, 45% were fractures–9% of the operative procedures performed were external fixations, and 14% of were primary internal fixations (61). Of the patients treated onboard the Comfort, 69% were adults, and 26% were children (61). The USS Bataan supported the USNS Comfort, arriving within 12 days of the disaster (47). Personnel onboard the USS Bataan treated 47 surgical patients, 87% of whom had sustained injuries related to the disaster, conducting a total of 109 surgical procedures (61). Of their total caseload, 72% of the patients were adults, 21% of the patients were children, 41% of the total injuries sustained were fractures, and amputations made up 3% of the operative procedures (61). The most active specialty involved in patient encounters were Trauma and Orthopaedic (T&O) surgeons, primarily treating 55% of the patients on both the USS Bataan, and the USNS Comfort (61). Furthermore, dental and medical professionals of the 24th Marine Expeditionary Unit (MEU), of the USS Nassau, treated over 100 Haitians (2). The care provided at sea, was supported on shore, through the opening of an aftercare facility (9). Within the Port-au-Prince area, infantry units from the 82nd Airborne Division, “helped facilitate emergency medical services by establishing trauma care facilities, delivering critical medical supplies, providing security at aid stations, and facilitating the transfer of injured patients” [(2), p. 62] to international facilities.

**Figure 6:**
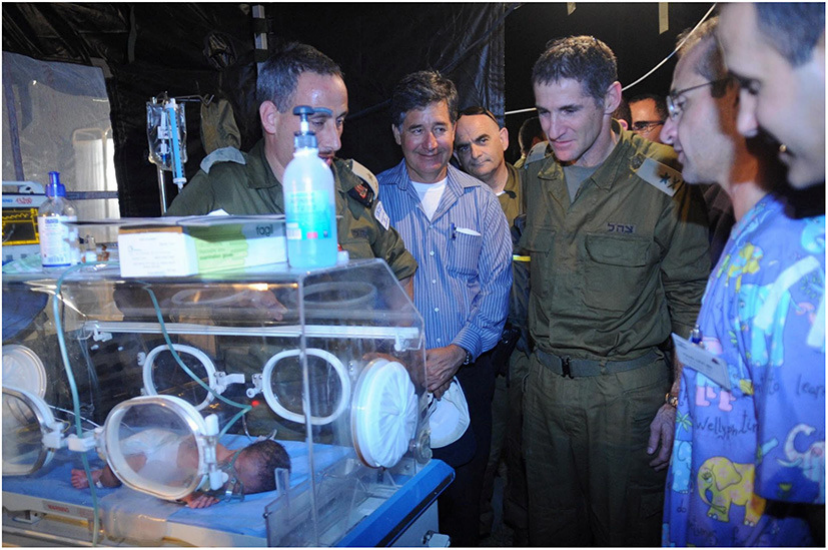
OC Home Front Command, Maj. Gen. Yair Golan, pictured here on a visit to the IDF Field Hospital in the premature baby maternity ward. After the devastating earthquake which struck Haiti in January 2010, Israel sent an aid delegation of over 250 personnel to help with search and rescue efforts and establish a field hospital in Port-au-Prince. Source: Public domain image, not in copyright. Available at: https://commons.wikimedia.org/wiki/File:Flickr_-_Israel_Defense_Forces_-_Head_of_Home_Front_Command_Visits_Aid_Delegation.jpg.

A number of other militaries contributed to the medical response in varying capacities. Colombia, France, Mexico and Spain also sent hospital ships, most of which were deployed for under a month (14). The Spanish ship, the Castilla, remained for a total of 64 days–28 more than the USNS Comfort (14). The vessel had capacity for 70 beds in total, including eight intensive care unit (ICU) beds (14). Medical professionals saw a total of 7,568 patients, reviewed initially at a land based mobile health unit, and conducted 104 surgical procedures (14). Both Canadian and Israeli military forces, utilised FFHs in the disaster response (28, 62), which are rapidly deployable treatment facilities. The Israeli military had previously developed an airborne field hospital model, that was structured to function in disaster settings (29). It utilised self-sufficient and flexible capabilities (29), with a total of 120 staff (62). Their workforce was composed of experienced and inexperienced personnel^10^, with the intention of facilitating knowledge transfer during relief efforts (29) (Figure 6). They also augmented work force capacity, by incorporating eight clinical staff from Colombia, which allowed them to run a total of four operating theatres (29). This unit initially functioned as a tertiary medical centre, until the USNS Comfort arrived (29). The Israeli Defense Force's (IDF) hospital was functional within 3 days of the earthquake (28), admitting their first patient at 10:00 a.m. on January 16th (63). The IDF offloaded the overburdened local health system, by dealing with patients who had suffered injuries directly pertaining to the earthquake. They treated 1,111 patients, admitted 737, and performed 265 operations (63, 64). In the first 3 days of operation, ~80% of presentations were due to traumatic injury (63). Of those patients admitted, 66% had sustained trauma, and of these, 46% had fracture injuries (64). The most active specialty was T&O, who conducted 83% of the operative procedures undertaken (64). In the case of the Canadian FFH, which arrived in Haiti after 17 days, the caseload encountered was predominantly patients (over 80%) who were not directly injured by the earthquake (28). During the 48-day deployment of the Canadian FFH, 151 patients received a total of 167 operative procedures (28). Of the operations performed at this facility, the overwhelming majority were inguinal hernia and hydrocoele repairs (28).

##### Civilian-humanitarian response

Of the civilian-based responses, the most comprehensive documentation was provided by *Médecins Sans Frontières* (MSF) and the International Federation of Red Cross and Red Crescent Societies (IFRC) (10, 27). The responses documented by both MSF and the IFRC, encompassed not only the initial emergency period, but also detailed efforts of the post-disaster response and re-development process. Further to this, the Cuban Medical Brigade (CMB) and academic institutions, participated in relief efforts—as well as medical professionals of the Haitian diaspora (14).

MSF, the “largest provider of emergency surgical care” during the humanitarian intervention [(14), p. 73], had staff in Haiti at the time of the earthquake. Therefore, their initial response began within hours (27). This included, evacuating patients from existing units, searching for appropriate facilities to continue care, and assessing new casualties—which sometimes had to occur in office spaces (27). In the early stages of the response, finding specialist treatment for the complex trauma patients, was imperative. MSF facilitated this by transferring patients to the Dominican Republic (DR) by helicopter (27). Although support staff arrived within 18 h of the disaster, difficulties were still encountered. Notably, the lack of available emergency medical equipment, such as drills, for use in burr hole procedures (27). This was compounded by logistical issues, with 11 out of 17 flights bringing personnel and supplies, having been diverted in the first 6 days (27). This meant deliveries had to arrive by road, from the DR, resulting in substantial delays (27, 41). Despite this, during the first 20 days of the emergency response, MSF clinicians had undertaken 1,300 operations, 140 of which were extremity amputations (27). The majority of surgical procedures conducted in the first month, were wound debridement and orthopaedic interventions (14). Early on in relief efforts, MSF partnered with the Renal Disaster Relief Task Force (RDRTF)—enabling a fully functioning dialysis centre, to be established 5 days after the earthquake (14, 65). Four and a half months into the response, 19 health facilities^11^, with over 1,000 available beds, were being managed by MSF; over 170,000 patients had been treated^12^, and 11,748 surgical procedures had been conducted (27).

The response of the Dominican Red Cross was immediate, dispatching a volunteer cadre across the Haitian border (10). The IFRC deployed two mobile field hospitals, and four basic healthcare units (11). They also managed a further 41 mobile, and five fixed health facilities (10, 11). By June, they had treated 95,500 patients, the majority of which received care for “non-communicable diseases and everyday emergencies” [(10), p. 34], and conducted a total of 1,339 surgical procedures. Additionally, they had extensive community-based healthcare programmes, reaching over 9,000 patients through these outreach initiatives, and provided vaccines to 150,000 Haitians (10). The CMB, who had an established presence in Haiti since 1998, had 330 healthcare personnel in the country at the onset of the crisis (14). They were able to begin assessing patients within 90 min, and conducted 1,000 emergency medical reviews in the first 24 h (14). They had access to a broad range of specialties, and 14 operating theatres—their staff also included colleagues from Canada, Chile, Colombia, Spain, Mexico and Venezuela (66). By January 27th, the CMB had delivered care to 14,551 patients and conducted 1,252 surgical procedures (66)—throughout the response, over 1,500 personnel from CMB were involved in delivering healthcare (14). Other specialised medical organisations that contributed to the emergency response, included Merlin and *Médecins du Monde* (11)—but there was little discussion of their activities. Moreover, it was noted that an initial restriction in capacity to provide post-operative care, meant that only a few life-saving emergency surgical operations could take place in the immediate post-earthquake period (11).

Six academic medical institutions from Chicago, participated in the medical response (14). By April 1st, the Chicago initiative had deployed 158 volunteers for minimum periods of 2 weeks and were integrated into established medical NGOs (14). The Harvard Humanitarian Program, led by “Partners in Health”, a non-profit organisation, operated across nine medical locations (14). By June 19th, 50 medical and surgical professionals had been dispatched along with medical, surgical, and anaesthetic supplies (14). During the initial 9 days of the response, the University of Miami's “Project Medishare” hospital, was based inside the UN compound. Its 250-bed capacity was staffed by only 12 individuals, and had no critical care or surgical capabilities (14). This was then transferred to a four-tent facility at MTPP, manned by 220 volunteer workers, rotating over 7-day intervals, with capacity for a specialist spinal care unit (14). This collaborative institution, utilised robust administrative and logistical capabilities, “coordinating flights to transport medical staff, supplies, equipment and victims between Haiti and the United States” [(14), p. 49]. The contribution of diaspora Haitian medical professionals was briefly discussed. Sixty clinicians from the Association of Haitian Doctors Abroad, were integrated into the *Hôpital d l'Universite d'Etat d'Haiti* (Haiti's University and Educational Hospital—HUEH) workforce on January 16th, setting up the initial emergency care unit at the institution (14).

MSF worked closely alongside Haitian clinical staff, in delivering medical assistance throughout the response (27). Although initially, recruitment issues were noted, in total they employed 2,807 Haitian staff—over 90% of their workforce—including doctors, nurses, administrators, project coordinators, drivers and logisticians (27). Furthermore, MSF also considered developing medical skill sets during the disaster response, an analogous approach to that of the IDF. The civilian organisation aimed to work with Haitian clinicians to “reintroduce… techniques” that they had been unable to utilise, due to a lack of surgical equipment [(27), p. 17]. The IFRC, similarly experienced issues recruiting staff in the early phases of the response—however, by June 2010, were employing over 1,000 Haitian national staff (10). A further example of local involvement, was the CMB's utilisation of Haitian medical students and interns—who were completing their training in Cuba at the time of the disaster (66). Humanitarian agencies, more generally, were noted to recruit large numbers of Haitian doctors, paying “salaries several times (higher than) their pre-disaster incomes” [(14), p. 39]—which, although a common practise in humanitarian responses, has detrimental implications for the host nations health systems and recovery.

##### Haitian-humanitarian response

An estimated burden of 30,000 genitourinary injury cases was reported in the Haitian peer-reviewed literature (67). In correlation with foreign opinion, better coordination was deemed essential for the implementation of “mobile disaster-specific medical units with tools to help disaster specific injuries—such as crush syndrome and spinal cord injury after earthquake—are paramount to improve patient survival” [(67), p. 6]. The same report, highlighted the new disaster-related medical and social needs affecting a significant proportion of the population, requiring long-term treatment and infrastructure.

**Figure 7:**
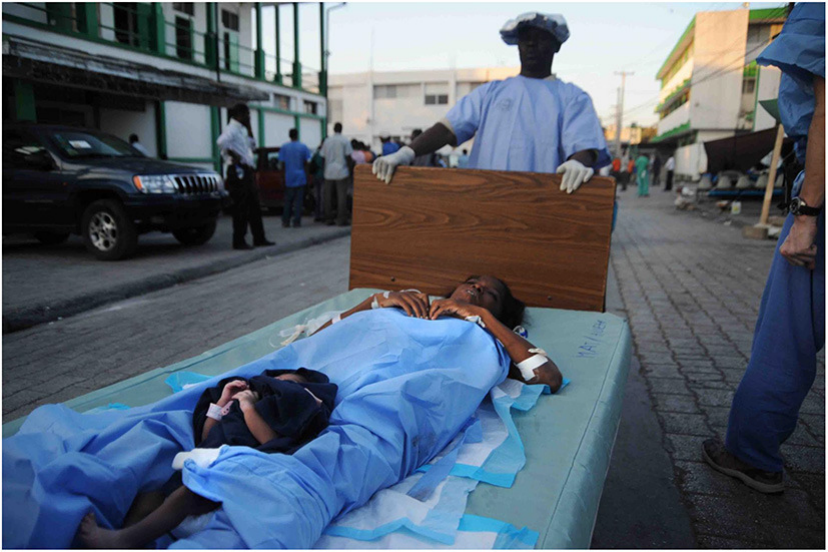
Medical personnel transport a Haitian woman and her new-born son to the post-operating room at the University Hospital in Port-Au-Prince, Haiti, January 20, 2010. VIRIN: 100120-n-6070s-016. Photograph: Petty Officer 2nd Class Justin Stumberg, USN. Source: Public domain image, not in copyright. Available at: https://commons.wikimedia.org/wiki/File:Newborn_baby_%26_mother_moved_to_post-op_at_University_Hospital,_Port-au-Prince_2010-01-20.jpg.

The Department of Anaesthetics at HUEH reported on this transition process. In 2012, an evaluation conducted after a substantial number of humanitarian NGOs had left Haiti, found the burden of restructuring and development while attempting to uphold quality of care, taxing and slow. The lack of sufficient standard operating procedures, human resources, and clinical staff, caused disorganisation in the delivery of surgical care—further perpetuated by healthcare providers leaving Haiti, or acquiring relatively well-paid NGO employment (68) (Figure 7). The need for central governance was highlighted as a potential solution to improving the delivery of safe patient care: “with the efforts of our health authorities, the wealth of our human resources, and the help of external cooperation, we can achieve the interdependence that is our mark of respect for ourselves and our patients, in order to ensure the safety and quality of care that we desire” [(68), p. 21].

##### Medical response: Additional themes

###### Readjusting healthcare priorities

The healthcare needs of the Haitian population evolved as relief efforts matured, and the priorities of the humanitarian mission had to change to mirror these (9, 27, 29, 40, 59). The IDF, and both the Canadian and US military, recognised that patient levels and presentations altered as the response continued (28, 29, 40, 59). The Canadian FFH had noted, that during the disaster response, the majority of their operative caseload was for pathologies unrelated to the earthquake (28). The USAF SPEARR team commented that their usual mission of providing immediate “resuscitative and stabili(sing)” care [(40), p. 63], was not applicable, due to fewer patients presenting with untreated acute injuries by the time they had arrived. The IDF readjusted staff assignments, unit organisation, and hospitalisation policy, as patients with less urgent medical needs began to present to the hospital (29). Once patients had received treatment, they were transferred to local facilities for ongoing post-operative care—which facilitated patient flow, and sustained delivery of medical aid to disaster victims (29). This process was mirrored aboard the USNS Comfort, who transferred patients to medical facilities run by the GoH and NGOs, for ongoing care (59). By February 28th, the emergency patient load had decreased, and no further patients with earthquake related pathologies remained onboard the USNS Comfort (59). As the medical capabilities of Haitian and NGO managed facilities returned to pre-earthquake capacity, the delivery of care provided, was transitioned to their jurisdiction (9, 59). During the crisis, the healthcare needs of the population encompassed two phases (27). In the first phase—during which, surgical priorities shifted from life to limb saving—surgical capacity was expanded significantly (27). This patient cohort consisted, predominantly, of those with neglected wound infections. The second phase occurred, because of hospital facilities being saturated with patients recovering from their injuries and operative procedures (27). During this phase, clinical space needed to be created, and an increased number of hospital beds was required for longer term patients (27). MSF was able to reinforce provisions for non-earthquake related pathology, by transferring these patients to other facilities (27), in a similar manner to their military counterparts. They also began consolidating medical facilities, following the overall shift in clinical priority, directed by capacity and capability at other NGO and GoH run healthcare institutions (27). It was also noted that several rehabilitative units were established—particularly those that were able provide care to patients with traumatic spinal cord injuries^13^ (SCI) (14).

###### Addressing re-development

Transitioning from disaster response to re-development, was another prominent theme with regards to the disaster response (2, 9, 10, 23, 25, 27, 44, 55, 59). MSF and the IFRC, committed substantially to re-development projects (10, 27). Although military actors did not plan to participate in re-development efforts themselves, the Joint Task Force-Haiti (JTF-H) objective—as defined in the OUR mission statement (55)—was to support humanitarian action and provide foundations; from which, the GoH, USAID, and MINUSTAH, could undertake long-term recovery work (44, 55). In light of this, transition planning commenced shortly after the onset of the crisis, with USAID—alongside military augmentation—establishing a “Future Planning Cell” (9). It was noted, however, that there was an ill-defined end point to military operations, and a dearth of strategic guidance with respect to this (9). This, coupled with the GoHs “limited… capacity” [(9), p. 144], lack of consistent financial resources, and legal issues, led to delayed implementation of military handover plans. Finally, regarding the theme of transitional humanitarian activity, numerous stakeholders utilised “cash-for-work” schemes, in a breadth of sectors, to “promote economic and political stability” [(23), p. 31], stimulate reconstruction, and facilitate long-term development. These were largely successful (25), despite reports of issues with establishing guidelines and equitable payment processes, which led to tension amongst the Haitian population and competition between programs (23).

### Resources

Within hours of the earthquake, humanitarian aid and disaster response teams around the world began to mobilise. By the day after the earthquake, the UN had committed $10 million US dollars (USD) from its emergency response fund, and the EU committed €3 million euros, with its member states allocating an additional €92 million (16). By mid-February, the UN had requested $1.4 billion USD for the response (16). The United States pledged the largest relief fund it had ever provided for a foreign disaster, spending over $1.1 billion USD. Eventually, private citizens in the US would donate another $1 billion USD (23).

#### Civilian resources

Despite the massive amounts of funding and supplies sent to Haiti, some UN cluster leads, noted that they had not received sufficient resources. In fact, unequal distribution was a major problem, with some clusters receiving more than they required, and others—especially those clusters relevant to long-term redevelopment^14^—being relatively neglected (25). Furthermore, as disaster events are relatively uncommon, organisations providing disaster relief services are often chronically underfunded and understaffed. The huge mobilisation that had to swiftly take place, overwhelmed some of these groups (19). Additionally, many inexperienced organisations and even individuals, felt compelled to travel to Haiti to offer relief services. While this may have been well-intended, it greatly challenged the humanitarian structure. People arrived who were not self-sufficient, and did not have the proper training or capabilities to enhance the response. Beyond a kind of misguided altruism, there may have been other motivating factors pushing these inexperienced actors into Haiti. Disaster relief activities have high visibility, and provide an opportunity for organisations to increase their credibility to donors, and their ability to compete for funding (43). It is worth noting that this may well have contributed to the influx of relief organisations to Haiti (43).

Despite the massive influx of personnel, equipment, supplies, and money, the response was hindered by an inability to manage what resources were available. In the early days of the response, the ability to deliver materials to the places where they were needed, was lacking. Considerable resources converged in Haiti, but were not necessarily able to get to the points of greatest need (49). The presence of resources alone is insufficient; they must also be accessible and properly used. In the case of the 2010 Haiti earthquake response, some supplies were sent without the relevant equipment, staff, or logistical support to use them. Responders arrived without transportation, or the ability to communicate with affected parties^15^, and therefore, their other skills or resources were under-utilised (19).

The initial response often focused on “secure” areas, which left poorer regions with less access to aid. Some of the urban population relocated to rural areas, which, although decreased resource strain in Port-au-Prince, placed increased strain on host communities. This was further aggravated by the lack of humanitarian actors and aid distribution mechanisms in these areas (2), since humanitarian groups tended to base themselves in the capital. In some cases, the distribution of aid itself, caused additional needs; for example, geographic inequities in aid distribution, caused some affected individuals to leave what may have been more stable areas, to access needed relief. This is exemplified by people who moved to camps to access aid centralised there, thereby exposing themselves to increased population density, and its associated risks (1).

#### Military resources

Multiple branches of the US military responded to the earthquake, under the auspices of the JTF-H (2, 9, 19, 23). JTF-H rapidly deployed personnel and supplies, which was effective in saving lives and reducing suffering—but, came at the cost of efficiency (9). Aspects of the response included civil and public affairs groups, engineers, and medical teams. Military Sealift Command ships, such as the USNS Comfort, are in continuous operation, and so were able to respond to the disaster swiftly (51). The hospital ship has a 1,000-bed capacity, including 80 ICU beds, in addition to 12 operating rooms, imaging options including a CT scanner, a full laboratory, and an extensive blood bank (61). The Air Force also contributed medical response teams, and although these were less well-resourced than those of the USN, their ability to respond rapidly was commensurate. The USAF SPEARR teams deployed in the first days, attended the disaster with surgical supplies in backpacks, along with one pallet of additional equipment—including a treatment tent and portable generator (40)—and were able to access patients when other, less mobile teams, could not. Despite these early deployments, the overall medical response of the US military was hindered by insufficient medical personnel, staff training, and experience for a response of the magnitude required—as acknowledged in US military reports. There were no medical logistics or regulating officers sent initially, who are critical for ensuring medical supplies and equipment are sourced correctly, and available when needed (9).

Efficiency across the JTF response improved when a working group was established, that held daily discussions on inbound supplies, equipment, and personnel. However, this system was not in place in the early days of the response (9). Overall, the response was limited by its lack of definition. Its role, and therefore the responsibilities and authority of the organisation, was not evident in the early days. Lack of early situational awareness also limited decision making on priorities for the response, making deployment of personnel and equipment more challenging. Forces and supplies entered Haiti in an *ad hoc* manner, not according to formal needs assessments, planning, and distribution procedures (9). Issues with logistics and resource allocation are clearly shown in the example of water. Initially, the capacity to distribute water exceeded what was available. With the arrival of the USS Carl Vinson, a supercarrier that can house thousands, the opposite issue arose. They were able to produce a large amount of portable water, but did not have enough containers to deliver what they were producing (69). Other military teams were noted in military reports to have been assigned tasks, not because they were necessarily the right personnel for the job, but simply because they were already present in-country (47). Even so, the US military's massive influx of manpower and supplies were critical to life saving efforts. At its height, on January 31st, the JTF-H response consisted of 22,000 troops, including 7,000 based on land, with more than 33 ships and 300 aircraft (12).

### Needs assessment

Needs assessment in the disaster setting, provides vital information on the overall impact of the crisis, which can then be used to direct relief efforts and ensure efficient use of resources. It encompasses two separate, but related, processes: a rapid assessment used to guide the initial response, and a more comprehensive post-disaster assessment. A rapid needs assessment is critical to make sure responders understand the needs as they stand and develop. In Haiti, it was delayed by negotiations and attempts at consensus-building, rather than fulfilling its greatest mandate: to quickly assess needs so as better to guide the flow of relief. An initial assessment, one of 10 cross-sector surveys costing $3 million USD, did not release its results until February 25th, over a month after the earthquake (14). Additionally, it did not include an assessment of Haitian capacity.

#### Military actors: Needs assessment

US military actors also conducted their own needs assessments. For example, AFSOC conducted medical site services over 16 sites to assess medical assets (40). Assessors on the ground were able to gather the most useful information on the state of the disaster; however, it takes significantly more time to put these actors in place, and then obtain the information needed to guide the response (70). Therefore, immediately following the earthquake, the extent of damage was unclear. The initial response proceeded without awareness of specific needs, requiring myriad assumptions to be made to commence planning.

Daily assessments were performed by the JTF-H Information Operations team, and this information was provided to the JTF-H commander. Verbal orders were heavily relied on, which led to a lack of an audit trail and hindered force planning and tracking (9). Early difficulties in gaining situational awareness, clouded the determination of requirements and priorities, greatly complicating the delivery and distribution of manpower and supplies. In addition, without a clear needs assessment present, JTF-H adopted a “push” approach—meaning supplies and personnel were sent until the command said to stop (70). Having decided that there was no time to gather complete information about the status of airports and seaports prior to the initial push of relief, and in the absence of coordinated logistics command and control infrastructure, much material was sent to Haiti without detailed plans in place (9). JTF-H were able to supply relief quickly, yet without situational awareness and a needs assessment, these operations were not conducted as efficiently as they may have been. Later, with more resources present, and with improved situational awareness, they transitioned to a “pull” response—requests were made in accordance with needs, leading to increased efficiency and resource flow (70).

#### Civilian actors: Needs assessment

The difficulties posed by the lack of workable needs assessments, was also keenly felt by civilian humanitarian responders. For example, the small USAID team on the ground initially was quickly overwhelmed, and unable to develop a common operating picture of Haitian medical facilities (13). Data on conditions on the ground, and dissemination of this data—as well as monitoring the quality of aid—are essential for aid targeting and distribution. Although general information pertaining to the disaster was widely available, “detailed ground level information needed for the effective distribution of supplies was lacking” [(13), p. 9]. Many humanitarian actors expended enormous time and effort to amass needs assessment data, but they each developed their own methodologies and tools, making it difficult to aggregate data and gain a comprehensive picture of needs (71). Overall, need and capacity assessments were weak early in the response, and the absence of clear agreement on the parameters of humanitarian need, led to a breakdown in communication with partners—notably the UN and GoH (6). Information management was a major difficulty. Whilst this was meant to be run by the UN's Office for the Coordination of Humanitarian Affairs (OCHA), its small staff and budget, meant that NGOs were depended upon to achieve this, by reporting their findings through the UN's cluster system (19). However, some of these actors were not well trained or highly skilled. It took almost a month for needs assessment to be completed, and by then it was not considered useful, due to delays as well as concerns about methodological flaws (19).

In addition to this, the process was extremely time consuming, with the needs assessment format that some organisations had collectively adopted *a priori*, requiring 3 h to answer all questions, and producing outputs slowly. Results, therefore, took up to several weeks, making some of the results yielded unusable (14, 25). Decisions about donations and goods, were made under great time pressure and with little knowledge about local needs. Additionally, some assessment teams arrived late and “reinforced the… belief that local capacity was too minimal to be included in the international aid response” [(25), p. 23–24]. Overall, needs assessments lacked clear context and analysis of local capacity, and due to this lack of knowledge, “relief efforts and support programs were often unilaterally installed and enforced” [(25), p. 26]—without considering the resources, needs, and desires of Haitian people. Haitian civil society organisations were largely excluded in designing and implementing programs, as the false assumption was made that local capacity was limited prior to the earthquake, and therefore must be non-existent after it (25).

### Communication

“Information management, including in the health sector, appears to be one of the weakest points of response in past disasters. The situation is compounded by the proliferation of general actors as well as agencies addressing highly specific needs.” [(14), p. 111]

In any humanitarian response, communication is arguably the most important domain, as all other response domains will fail or succeed, based on communications (72). The destruction included the telephone lines, mobile phone circuits and the electrical grid—which led to oversaturation of limited satellite phones. Furthermore, there was minimal internet access, as the only undersea cable came ashore at Port-au-Prince, and this was significantly damaged (13, 14). Communication is inherently collaborative in nature, and so this section will analyse the interaction between civilian and military actors, during the disaster response.

#### Civilian and military interaction: Communication

The first issue was language. Most meetings were conducted in English, less frequently in French, and none in Creole (25). Very few of the foreign teams that responded to the disaster were able to communicate in French or Creole (14, 25, 73). Lack of ability to communicate in the language of the affected population, led to confusion about where and when aide distribution would be (25). More and more foreign teams arrived, needing interpreters, particularly for the medical response (39). The US military additionally pointed out the importance of local interpreters, as they also served to educate the responders about the Haitian culture (40).

Information gathering and dissemination, negatively impacted the medical response in Haiti as well. The “ability to pass timely and accurate information was as important as the availability of food and water” [(38), p. 60]. Multiple agencies, including Haiti's *Ministère de la Santé Publique et de la Population*, the Centre for Disease Control and Prevention, and the Pan American Health Organisation, established two systems for surveillance of infectious outbreaks. The data collected into these systems, came from multiple sources, was not standardised, and was of varying degrees of quality—which made interpreting and reporting outbreaks challenging (23).

The relief response in Haiti relied heavily on smart phones and internet for communication. This method of communication was a major issue when attempting to coordinate with the USN and US Coast Guard ships (39, 51)—where these modes of communication are not routinely used. This impacted the effectiveness of the hospital ships. Furthermore, in the context of the USNS Comfort, there was a breakdown in communication about the number and types of patients that it was able to receive, as well as casualty collection point information. Once patients were onboard, there was a delay in establishing how families could get information about their care (74). Additionally, terms utilised, such as “MEDEVAC”, had differing meanings between organisations, which created delays and inconsistencies in prioritisation of patient transfer (75). There were four large hospital ships that responded to Haiti, in addition to the USNS Comfort, and all used a different referral system. Each hospital ship did not communicate their admission criteria to each other either. The IDF circumnavigated the issue of medical miscommunication, by designing and implementing their own electronic medical records. As records were backed up on computers, loss of patient information and medical error were minimised (29).

### Coordination

Although there is overlap between communication and coordination, the process of coordination is distinct from simply employing effective communication. As one review put it, “coordination requires the existence of a set of principles, rules and decision-making procedures generally accepted by stakeholders” [(16), p. 150]. While these principles are generally well-established within an organisation, the interplay between various stakeholders proved to be the biggest obstacle in coordination of relief efforts in the 2010 Haiti earthquake response. It cannot be understated how the vast number of countries, militaries, and NGOs, responding to the disaster, played a role in the difficulty with coordination (2, 9, 44). This section will focus primarily on the coordination of efforts between civilian and military actors.

#### Civilian and military interaction: Coordination

Just 11 h after the earthquake, the IDF sent a medical team to conduct a needs assessment and make local contacts for coordination of supplies and where to establish their field hospital. Due to the rapid arrival of the IDF field hospital, they were rapidly inundated with patients, and were forced to serve as a coordinating referral centre for medical teams that were subsequently established in the area. The coordination with local and foreign medical teams was successful in increasing capacity (29, 63, 64). Within 2–3 days, multiple universities and NGOs were in Haiti, and working on coordinating patient flow—including collaborating with the US military to send patients *via* aeromedical evacuation to hospitals outside Port-au-Prince (74). This coordination required establishment of medical liaisons, who would physically travel to facilities to ascertain capacity and capability (28). When the US ships arrived—with intrinsic surgical capability—the field hospitals were, for the most part, well-established. A referral system was set up, so that local providers could send patients for triage to military medical teams ashore—patients were then transported to the ships for complex care (61). The arrival of the USNS Comfort brought with it a high level of surgical and medical capability. While only military surgeons were initially on board, personnel from NGOs were quickly brought in to reinforce capacity to conduct complex reconstruction surgery—which was much easier to accomplish on the hospital ship, vs. the FFHs (76). Military coordination was land based as well as sea based. The USAF set up an EMEDS system, based at Terminal Varreux. This site coordinated with the USNS Comfort to take patients that required long term care, and rehabilitation. They worked with the Haitian Ministry of Health, to coordinate patient movement to local hospitals and NGOs (40). In addition to the US hospital ships, four others arrived from Colombia, France, Mexico and Spain. Each had their own referral system and admission criteria, which led to confusion about coordinating patient movement (14). The IDF, and both US, and Canadian militaries, recognised the importance of appointing liaisons to physically travel between the facilities to coordinate referrals (28, 64). Exemplary coordination continued up until the point of departure, with the IDF ensuring patient hand off to appropriate medical and non-medical facilities (29).

Many NGOs contacted the military medical efforts to volunteer services. Both Project Hope and Operation Smile, had conducted missions with the hospital ship previously. Project Hope had an existing memorandum of understanding (MoU) with the USNS Comfort, which led to rapid integration (51). Go Team, another NGO, also had an MoU in place with USN Southern Command, which also greatly aided integration with the military (51). Operation Smile, faced difficulties in finding who on the military side authorised integration—and put extensive work into trying to support the military, with little success (51).

### Pre-existing policy

There were significant delays in response time to the 2010 earthquake, secondary to the pre-existing policy which was in place at that time. In general, previous policy frequently required approvals for resources to be accessed, and the need for these approvals led to delays in mobilisation (72).

#### Pre-existing policy: Military

Concerning this response, there was a considerable amount of high-level policy, which was either in need of updating or completely non-existent. Within the US military, this was particularly glaring. Only two Humanitarian Assistance and Disaster Relief (HADR) doctrines existed, and the general plan was outdated (13, 44). Within US Southern Command (USSOUTHCOM), the plans that existed, were created for the prior organisational structure, and had not yet been revised to reflect the recent restructuring (70). USSOUTHCOM, the joint military command responsible in the region, was the lowest staffed command in 2010, and its limited personnel led to diminished ability to respond rapidly and effectively (77). No formal guidance existed for the use of USN ships in HADR, and therefore plans in the Haiti response were modelled off casualty care plans, rather than HADR (61). In the initial response, the nearest ships were selected to respond, though this may not have been the best plan of action (78). The Oslo guidelines are frequently cited to help define governance, and they encourage the use of military assets in humanitarian efforts—though UN policy generally is not in favour of such collaboration (6, 13, 43, 79). To that extent, the US military system had policies in place to facilitate participation in the earthquake response, but much of their capabilities are intertwined with various domestic entities. For example, the Patient Movement System was designed for use by military beneficiaries, but is capable of other mission support. However, this requires it to be called upon by the National Disaster Medical System, and to remain under the coordination of US Transportation Command^16^ (33).

As the initial response ended, the US military and other actors, needed a protocol for exiting (43). This guidance was not established prior to the earthquake, but is necessary for the military to leave upon mission completion (47). Though rapid deployment is the military's greatest strength, dependency and expectation must be avoided, and because HADR typically leaves little time for policy establishment, it is imperative that this is established beforehand (13).

#### Pre-existing policy: Civilian

Poor or incomplete policy, contributed to a general lack of preparation for a disaster of this magnitude, a particular disappointment given the presence of the international community in Haiti for many years (9). In Haiti, at the time of the earthquake, was the UN's stabilisation mission—MINUSTAH. However, this was built to maintain law and order rather than to respond to a disaster. Furthermore, their central leadership was affected by the earthquake—significantly impairing their capability as a force (19, 32). Within Haiti, though NGOs such as MSF had taught emergency techniques in local hospitals, limited equipment and supply, led to an inability to practise and adapt these techniques (27). MSF also lacked a pre-formed plan to respond to an emergency of such magnitude (27). Intragovernmental US agencies, such as USAID and the Federal Emergency Management Agency, were also in need of policy improvement to combine their efforts, as their redundancies and lack of leadership contributed to delays (9).

## Discussion: Lessons learned

Medical disaster responses have enormous potential to shape the re-development processes that follow. It is essential, that humanitarian practise is guided by evidence, which can be gained through analysing previous relief efforts. The response to the 2010 earthquake in Haiti, remains one of the most complex and expansive humanitarian endeavours to date. Even more unique, was the huge response from military forces. In analysing the data pertaining to each of the priority domains, many “lessons learned” were identified—which should inform future disaster response practise.

### The humanitarian response: Lessons learned

The first point to discuss, which was predominantly raised by military actors, is that a clear transition strategy is required from the outset of the crisis response (47, 51). Namely, a timely transfer of the responsibility for medical provision, to the jurisdiction of the host nation and other local and international NGOs. It is essential that this process engages and supports the local government (14) and does not undermine or disempower them, as was seen in Haiti. Following on from this, the local population should be heavily involved in leading the response, and “instead of managing the crisis themselves, international partners should accompany and build the capacity of their counterparts” [(14), p. 141]. This will likely require the sacrifice of short-term efficiency and coordination, while focusing more heavily on strengthening local capacity—which leads to sustained improvements over the long-term. As noted in Haiti, developing medical capacity can be driven by disaster response efforts—which can highlight gaps in medical care that need to be addressed. Following the humanitarian response, the prognosis of patients who suffered SCIs in Haiti drastically improved. This resulted from early international appeals for support, answered by specialists and physiotherapists (14). The influx of specialist resources, as well as an expansion in capability with regard to early supportive care and rehabilitation, meant that those with SCIs had access to a more appropriate level of care (14). The result was that Haitian patients, who previously would have died, now had a significantly improved prognostic outlook (14).

Medical activities must be led by guidelines and local practise. In Haiti, issues arose due to insufficient understanding of “the standards of local care and processes” [(2), p. 64]—meaning that a number of patients received inappropriate procedural interventions, that could not be managed within the local health system. Additionally, any actors who engage in humanitarian relief activities, should ensure that they utilise appropriate clinical governance practises with regard to patient documentation, to enable comprehensive follow up of any disaster victims to whom they provide medical care. Furthermore, they should actively inform themselves of the working practises of the local health system, to safeguard patients from inappropriate surgical treatment that cannot be suitably managed post-operatively.

It is essential that foreign medical teams do not exacerbate the substantial burden already placed on local health systems (80). In Haiti, there were several instances where the actions of the international responders disrupted national capacity, including: the “poaching” [(14), p. 39] of local health professionals, introducing a cholera epidemic (14), and commandeering local health facilities (14). Not only does this behaviour cause excess strain on capacity of the host nations health services, but it risks generating parallel health systems that weaken local infrastructure (81). To combat this, adequately trained personnel should be deployed during the early stages of the response (77, 82). Additionally, if medical infrastructure becomes so stretched that patients require extrication abroad, evacuation options need to be established, including for special patient categories (33). This option should only be a last resort, with preference given to strengthening local capacity. Furthermore, oversight over international patient evacuation, must remain with the national authorities of the host nation (14).

Collaboration between local, international, and military actors, can augment medical capacity during emergency relief efforts (64). This can be facilitated by fostering relationships, either prior to crises occurring—through interagency training and exchange exercises (9, 71); or during emergency efforts—by utilising an integrative FFH framework (64). These FFH units should be prepared to treat a range of pathologies, maintain flexible capabilities that are not tailored according to anticipated activity (64), and be able to support the fluctuating medical requirements of the host nation (63). This will support local health systems, a fundamental requirement when the response must be constantly altered according to the health needs of the host population (14).

### Resources: Lessons learned

The affected country's government is best placed to prioritise the flow of resources to reflect changing needs, as the disaster response evolves. As noted by the US military, their approval is an important endorsement, and has the additional benefit of decreasing complaints of favouritism, when this prioritisation is undertaken by a third party. In the face of a massive disaster, this will present a challenge for any government. For low- and middle-income countries, where there is less adequate infrastructure, personnel, and expertise in place—this task may become overwhelming. This suggests a role for an international organisation, to support the affected government in planning and coordinating transport of resources, that is deferred to by the international community in future disaster responses (43). Regional governmental agencies, such as subsidiaries of the UN, are well placed to fulfil this role.

Information is critical for deployment of resources. If the needs of the affected population are not identified and tracked, and the processes governing distribution of resources are inadequate—then knowing what additional resources are needed to effectively source and deploy aid, becomes next to impossible (83). In the early days of the response, logistics mechanisms were overwhelmed by the influx of supplies—some of which contained useless or complicated equipment, that had to be sent back. This wasted time and resources, and limited the space available for arrival of supplies which were acutely necessary. Preparation and planning for the in-country situation is essential. Those with roles in planning and policymaking, must take into consideration that the actual environment, may be significantly different to what is predicted. Information about the current situation on the ground, is essential to ensure that the correct human and material resources are sent to aid the disaster response. In many situations, not all the information will be available in the first hours and days. Forward scout teams may be sent to the affected area to analyse the impact of the disaster. They can provide information on where humanitarian actors may establish themselves, giving consideration to responder safety, and how to set up logistics to maintain self-sufficiency (80). Additionally, in areas that are known to experience frequent disasters, emergency supplies should be stockpiled, so that they may be easily accessed and dispersed in the immediate aftermath of a disaster (51).

Even organisations with extensive experience in Haiti were challenged by the scope of the response, and the unprecedented amounts of donations they received (27). Challenges included: the high financial cost of flying in materials, the bottleneck of the airport, a lack of electricity in hospitals in the early days of the response, a lack of water or food for patients, a lack of local knowledge of reconstructive surgery—due to the lack of equipment necessary to teach these techniques pre-earthquake, a lack of physical therapy, and a lack of psychiatric capabilities^17^ (27).

The military has a huge scope of capabilities that can be leveraged during a disaster response, including vertical lift, logistics, communication, and emergency and trauma healthcare. Furthermore, they possess the capability to deploy these assets quickly, in comparison to most civilian organisations (13). While the military can offer very advantageous equipment, whenever possible, locally available resources should be used. This helps to protect the local economy, so that it can continue to function after relief operations conclude (13). In the case of Haiti, the US Navy and Army were better able to capitalise on existing relationships in the region, than its Air Force. This was in part, due to the rotational nature of the Air Force's contractors—who relied on short-term, rather than long-term, partnerships (84).

A successful aid response requires more than good intention or boots on the ground; it requires the presence of people with the skills required to accomplish needed tasks, and the delivery and distribution of the supplies they require to do so. Incorporating adaptability into any team's structure is critical so that, especially early on in a response when there are still many unknown factors, operations may be adjusted to best provide needed services after arrival (62). This is true of all responders, though is exemplified by medical response teams, who must deliver care in accordance with the pathologies of presenting patients; this will greatly affect the number and type of personnel, supplies, and equipment necessary to run a health facility (62). Flexibility, in terms of both personnel and structure of a field hospital itself, are essential to a team's success. After the situation and its corresponding needs are better understood, priority areas can be identified and subsequently reinforced with additional supplies and staff. This idea of a “resupply”, based on actual needs, can be built into policy in the planning phase—as has been reported by IDF planners, who suggest this should occur ideally four to five days after arrival (64). Integrating medical units into the response early on is essential, and training these medical units to provide services in low resource environments, will ensure they can respond—even if the disaster has severely limited the resources available in the early days (52). Military capabilities, as discussed above, can also be advantageous to the medical response: they have medical personnel, equipment, and supplies, as well as the people and equipment to transfer patients and necessary materials (33).

The ability to monitor the number and potential contribution of medical teams in a disaster response is also essential. This requires administrative, financial, and logistical expertise, as well as medical expertise. This was challenged in Haiti, due to the large number of responders without sufficient experience or potential for meaningful contribution, who flooded into the country. Humanitarian medical responders, must also take care that their actions do not further disrupt the functioning and rebuilding of the affected countries. For example, large numbers of Haitian physicians were recruited by humanitarian organisations and offered much higher salaries than what they could earn by staying in Haiti. On a systems level, such actions can further deplete the affected nation's medical institutions and potentially weaken recovery efforts (14).

Needs, post-disaster, change as the response progresses. Immediately after a quake, medical needs are dominated by trauma. Later, medical issues arise that in most cases, could have been treated by the affected area's health system, were its infrastructure not damaged. Finally, infectious disease control, rises in importance. Healthcare relief can be optimised by transferring patients to the facilities where they can be best served. For example, high acuity patients can be sent to tertiary medical structures, while primary facilities can take care of a larger volume of patients with less acute needs. Different medical teams may have access to different personnel, supplies, and equipment. Pooling these resources, and distributing them to where they are most needed, optimises the reach and efficacy of care provided (83). This did occur in some cases during the 2010 earthquake response, for instance, nurses and medics were in short supply and could transfer between groups as necessary (83). The Red Cross also had supplies which were distributed between FFH (83).

In responding to a disaster, especially of the magnitude of the 2010 Haiti earthquake, hospital beds are a finite and precious resource. Maintaining bed availability for urgent treatment must be considered early in the response phase. This may be facilitated by taking discharge planning into account even early on, when bed availability is higher, and by creating temporary, lower acuity centres, where stabilised patients may be housed to free hospital space for those with higher acuity needs (74). Standardisation of record keeping among medical responders, would also be of benefit. Electronic medical records, help improve medical accuracy, by reducing the likelihood of information loss and gaps in continuity of care (29). This holds true in a massive disaster scenario, especially when patients can be transferred to medical teams of different countries, and there is a high amount of provider turnover (29).

Haiti's medical infrastructure was inadequate to its population's needs prior to the earthquake. Responders began treating conditions that had clearly existed *a priori*. While this may have been because the hospital that patients would have presented to had been destroyed in the quake, in some cases humanitarian actors were providing services that had not been previously available. While the humanitarian principle of humanity dictates that “human suffering must be addressed wherever it is found” [(85), p. 2], future responses could benefit from clearer goals at their outset based on the level of pre-disaster infrastructure (22).

People around the world donated to relief efforts in the aftermath of the earthquake—the American Red Cross alone, raised almost $500 million^18^ USD (86). This huge upswell of concern and support, however, could have been better leveraged. One suggested method, is to publish information on contacts that NGOs and donors, including private companies and private citizens, must reach out to about donating materials to response efforts (69). Donors may earmark funds for certain initiatives or aspects of relief efforts, in general they are within their rights to do so. However, certain clusters, including those responsible for indispensable redevelopment projects, can end up with comparably less funding (25). It may be beneficial to establish a financial system where some redistribution is permitted between clusters, so that discrepancies between cluster budgets and available funds are minimised (25). When funding is sent to implementing partners, consistent and continued assessment and monitoring, is extremely important to ensure that funds are being used appropriately and efficiently, and that the affected population is receiving the maximum benefit from designated funds (25).

### Needs assessment: Lessons learned

It is difficult to attain both accuracy and speed, when conducting post-disaster assessments. In this case, rapidity must be valued, and some accuracy neglected to achieve it—initial “rapid” needs assessments must fulfil the dictates of their name, and so speed should prevail over perfection. The aim must be having the right information in time, rather than perfect information too late—although in the case of Haiti, even the latter was not achieved (25). Humanitarian actors must standardise needs assessments. Inconsistencies in methodologies and tools, hamper efforts to build a comprehensive understanding of activities and needs on the ground, leading to the duplication of efforts and wasted resources. Lack of standardisation creates both “too much and too little data” [(71), p. 1107]. By creating better systems for data gathering and sharing, responders can work together more efficiently, and more successfully synthesise their information to prioritise needs and direct resources. Indicators must be chosen and followed by all data gatherers; this latter action was lacking in needs assessments conducted in Haiti. Once obtained, assessments must be followed by decisions that consider existing capacity, observed needs, and practical constraints. Information management is critical, because an excess of unstandardised data, requires inordinate effort to turn into actionable information. The priority is to gather timely information for the purpose of collective strategic planning, and to this end, mutual dedication to an agreed set of standardised indicators is key (14). Open-source information systems, that emerged during this crisis, could be utilised to store the findings of such assessments—enabling all stakeholders to have access to this key resource.

Future responses must rely on improved needs assessments and stronger linkages between the humanitarian community's strategic and operational levels, to target humanitarian assistance more strategically. This could have reduced population movements and avoided additional needs and vulnerabilities, which arose later in the response (6). Importantly, needs assessments should be expanded to better understand context and capacity. Awareness of local capacity is imperative, and should be highlighted in needs assessments and given adequate consideration—otherwise civil society and the desires of the populace, may be ignored (25). Language has been highlighted as one reason for the lack of participation of local NGOs in the cluster system, but as suggested by one report, OCHA should undertake an assessment to better understand why this occurs (25). As per that same report, if context and needs assessments had been done well, “it would have been clear that local capacity was available and… the necessity to integrate… civil society in the response could have been identified” [(25), p. 30]. The post-disaster needs assessment should include information about physical and human damage inflicted by the disaster, financial information on the cost of reconstruction of physical damage, the value of income and services lost because of the disaster, and the impact on the affected population (14). These assessments should be supported by the international community, but should be requested and led by the affected government. In the case of Haiti, a formal request was not made until February 16th (14).

With regards specifically to the medical system, it is known that case mixes encountered by medical relief providers will likely differ based on the type of disaster—for example, more surgical or orthopaedic trauma cases after an earthquake, vs. more medical cases after a famine or typhoon. However, to optimise the response, more complete information about the needs on the ground is still required. From the experiences in Haiti, as well as Nepal, not all of this information is available to the local populace in the hours and days after the incident (80). A rapid needs assessment team, or in the case of the military, a forward scout team, can provide extremely useful insight by travelling to the disaster site and obtaining first-hand information, upon which to base decisions. The military's forward scout teams in particular, are logistically self-sufficient and can perform situational analysis based on disaster impact, time after disaster, and disaster type—as well as pick locations for deployment based on safety, accessibility, and size (80). Some needs are predictable: after reviewing the patient presentations seen aboard USN ships engaged in three earthquake responses, they noted that complex musculoskeletal injuries comprised an overwhelming majority of the disaster-related conditions they saw and treated, which can help future relief missions in determining, if not the supplies and capacities needed for the entirety of the earthquake response, at least those needed for the presentations the USN ships are likely to see (61). Limitations are similarly predictable, the speed with which responders are able to be deployed^19^ will be a factor in what cases they can manage, and this must be considered during planning. This idea can be extended to any organisation involved in early disaster response: the required capabilities that were noted in the early days, prior to rapid needs assessment, can be sent initially—with the understanding that improved situational awareness should guide further disbursements of equipment and personnel. Even without a needs assessment to guide action, the conditions under which any field hospital will operate must be anticipated, and planning conducted accordingly. A large number of NGOs are capable of providing basic care, and this need is predictable when responding to a disaster like an earthquake. Fewer organisations are capable of deploying a full-service field hospital, but organisations with this greater medical capacity may learn from the experience of the IDF, by sending self-sufficient, multidisciplinary teams in the initial response—when even a rapid needs assessment is not complete. This will add significant value to the overall medical response (64).

### Communication: Lessons learned

The response to the newly employed open-source information sharing systems, used during the 2010 Haiti Earthquake, was predominantly positive—however, some drawbacks were noted. The chief complaint about the data shared on these platforms, was that there was too much of it, and navigating the data to determine its relevance, was time consuming. This balance of rapidity vs. quality, ended up favouring rapidity. As the search and rescue efforts were relying on quickly translated messages, precision became less important than responsiveness (87). In some circumstances, the sheer volume of responses overwhelmed the crowdsourcing volunteers that worked on translation. For the military, the bandwidth needed to effectively use the internet, was not available on any of the US military ships. Besides the aforementioned overflow, of perhaps irrelevant information, and the bandwidth needed to run social media websites, the open-source sites had potential for misuse and abuse to include cyberattacks (87). This was not an issue in the 2010 response, but in future disasters, these freely open sources may make rescuers vulnerable, as the Global Positioning System (GPS) coordinates will be widely known. Also, in the current landscape, the potential for these sources to be used for spreading disinformation needs to be addressed (38).

### Coordination: Lessons learned

The foremost lesson learned, and action plan for future disaster relief operations, was the lack of training. There were internal and external complaints about the US military having a lack of expertise and experience in humanitarian and disaster responses. The UN and NGOs, recognised that they would benefit from cross training with the military as well (26, 47, 51, 78, 88). From these experiences, it was recommended that protocols and priorities should be established between military and civilian actors, and cross training should occur—so that coordination and communication during a disaster would be enhanced (83). Additionally, the US military recognised the need for pre-established plans, and HADR rules of engagement that were scalable (77).

Despite the vast number of medical teams in Haiti, there was not a centralised method of triaging and coordinating patients. That burden fell to the individual field hospitals and hospital ships. One recommendation for future disasters, would be to have centralised triage, managed by the UN's Disaster Assessment and Coordination system, which would ideally optimise resources (63).

It is important to mention that a major contributing factor, to the failure of coordination of relief efforts, is the marketised nature of humanitarian aid (89). The top-down structure of organisations (90), means that ear-marking of projects and “cherry-picking” of causes (91) has resulted in a competitive “market”, whereby initiatives are chosen for their visibility—rather than actual merit (89). Money and resources are gathered, but remain as mere capital, rather than being translated into useful areas for development and production (90). It follows, that centralisation emerges as a fundamental aspect of creating a global aid landscape that will seek to address the needs of the affected nations, and avoid “duplication, waste, incompatible goals, and collective inefficiencies” [(89), p. 17]. Furthermore, it is worth noting that the fundamental humanitarian principles of neutrality and impartiality, complicate military engagement during humanitarian response efforts (92). Both issues need to be addressed if additional steps are to be taken towards improving coordination.

### Pre-existing policy: Lessons learned

In future disaster responses, it is critical that logistics, staffing, and training standards be established, such that responders can do so appropriately (19). Were it not for previously established relationships, which allowed for deviation from policy, there may have been more substantial issues with the response (9). In the future, the overarching recommendation is that, if the US Department of Defense (DoD) is going to continue to have a role in HADR, they need a dedicated HADR chain of command (9, 43). By creating this, there will be a greater group of commanders, with the skills and training to lead in these situations (9). Because air support is so critical early on for transportation and logistics, a predefined role would be crucial moving forward—as the guidelines in 2010 were thought to be ambiguous (26). No one can debate the US military capabilities regarding command and control, communication, and logistics, as they are unique assets to HADR (43). A concrete and well-defined set of pre-existing policies, supported by a set leadership chain, would enable rapid response.

The influx of large numbers of international actors, has been a recurring theme throughout this study—especially those without the appropriate skills and expertise to be able to meaningfully contribute (11, 14, 19). This was not unique to civilian organisations; it was noted that the extensive US military presence “[hindered] the arrival of aid” [(20), p. 4]—with excessive numbers of non-medical DoD staff having been deployed initially, “[delaying]… medical assets reaching Haiti” [(33), p. 1130]. It is clear that there is a need for improved oversight and governance practises, with regard to organisational participation and conduct in humanitarian relief activities. Current regulation of international organisations, as well as mechanisms for maintaining accountability, are inadequate (93)—this was exacerbated in Haiti, by high levels of corruption (94). Expecting the institutions of the nation affected by crisis, to govern these processes, whilst monitoring the standards of those participating in the response, is unrealistic. International consensus should be reached on guidance and practise, with the aim of increasing standards and quality (25)—and both civilian and military stakeholders should contribute to their development. Once acceptable standards have been developed, the entire international community holds responsibility for safeguarding them. Ultimately, oversight for upholding these standards should remain in the hands of a civilian body. What this responsibility looks like, and to whom it will fall^20^, requires further investigation, and importantly sector-wide agreement.

## Limitations

The coordination and effort required to conduct research during active humanitarian crises is a significant undertaking. Data collection will always be secondary in the acute disaster event, and the priority of actors, correctly so, is to provide emergency aid to the affected population. This may result in “missing data” when conducting an evaluation, such as this current study. An understanding of the geopolitics and donor influence is required to decipher the agendas of both civilian and military organisations, that engaged in providing assistance. This information is not always readily available or widely publicised, which has implications for the research process, and the narrative of the literature disseminated.

Another limitation, is the large volume of eligible data available for analysis, despite the rigorous exclusion criteria. It is inevitable, even with thorough and systematic reviewing of the data, that some information may not have been captured. Additionally, alterations to practise, made by organisations since the earthquake, may not have been included.

Finally, the most significant limitation, is the lack of inclusion of the Haitian perspective in the available literature. It is essential that future research seeks to include and amplify the academic contributions and expert opinion of Haitian entities.

## Conclusion

It is clear, through this review, that the many stakeholders involved had varying opinions and perceptions of the same events. Despite this, the medical disaster response can largely be considered a success.

Future disaster responses must respect the doctrine of national sovereignty, and must not be imposed upon nations in severe distress. International actors must ensure operations are both inclusive, and empowering of host nations, so that they are able to take a leading role in relief efforts. The humanitarian community needs to direct attention towards developing international guidelines, setting a gold-standard for disaster response practises, and regulating the actors involved. Finally, great emphasis must be placed on the importance of fostering strong relationships between humanitarian actors, both civilian and military—which is critical in preventing organisations from “competing, rather than collaborating, to save the most lives” [(1), p. 127].

No modern disaster has yet been as devastating as the 2010 earthquake. Given the ongoing climate crisis, as well as the risks posed by armed conflict (95–98)—this will not remain the case indefinitely. Just as disaster responses influence post-disaster re-development, a nation's pre-disaster capability will influence any disaster response that becomes necessary. Low- and middle-income countries are at greater risk of experiencing natural disasters^21^ (100, 101) and the outbreak of armed conflict (102, 103), and simultaneously have health systems and national infrastructure that is less able to withstand the additional burden created by such events (100, 104). In pursuit of health systems strengthening and disaster preparedness, the international civilian and military medical community should seek to form strong and enduring partnerships with those nations most at risk.

## Data availability statement

The original contributions presented in the study are included in the article/supplementary material, further inquiries can be directed to the corresponding author/s.

## Haiti Disaster Response – Junior Research Collaborative (HDR-JRC)

Robert B. Laverty, Carlie Skellington, Carolyn Judge, Clara Hua, Elizabeth Rich, Rathnayaka M. K. D. Gunasingha, Peter Joo, Sarah Walsh, Tahler Bandarra, Tesserae Komarek, and Nava Yarahmadi.

## Author contributions

MA, MJ, and TW: study design, data analysis, writing, and critical revision. GC: study design, data analysis, and writing. MB, LM, SA, and RH: data analysis and writing. RL, CS, CJ, CH, ER, RG, PJ, SW, TB, and TK: study design and data analysis. NY: data analysis and manuscript revision. All authors contributed to the article and approved the submitted version.

## Conflict of interest

The authors declare that the research was conducted in the absence of any commercial or financial relationships that could be construed as a potential conflict of interest.

## Publisher's note

All claims expressed in this article are solely those of the authors and do not necessarily represent those of their affiliated organizations, or those of the publisher, the editors and the reviewers. Any product that may be evaluated in this article, or claim that may be made by its manufacturer, is not guaranteed or endorsed by the publisher.

## Author disclaimer

The opinions or assertions contained herein are the private ones of the author/speaker and are not to be construed as official or reflecting the views of the Department of Defense, the Uniformed Services University of the Health Sciences or any other agency of the U.S. The views expressed in this paper reflect the results of research conducted by the author(s) and do not necessarily reflect the official policy or position of the Department of the Navy, Department of Defense, nor the U.S. Government. I am a military Service member [or employee of the U.S. Government]. This work was prepared as part of my official duties. Title 17, U.S.C., 105, provides that copyright protection under this title is not available for any work of the U.S. Government. Title 17, U.S.C., 101, defines a U.S. Government work as a work prepared by a military service member, or employee of the U.S. Government, as part of that person's official duties.

## Footnotes

^1^For detailed historical discussion of the Republic of Haiti, please see texts by Moore (1) and Vialpando (2).

^2^Following the primary earthquake, a minimum of 52 aftershocks were recorded with a magnitude of at least 4.5 (6).

^3^This is the official figure reported by the Haitian government (7)—although, this number is disputed. Others estimate the death toll to be around 160,000–230,000 (2, 8, 9).

^4^Chief of Surgery at *Hôpital de l'Universite d'Etat d'Haiti*, Haiti's tertiary referral hospital, during the 2010 earthquake response.

^5^Director at the *Centre d‘Information et de Formation en Administration de la Santé* (CIFAS/MSPP), the Haitian “Centre for Health Administration, Information, and Training”.

^6^Civilian Professor at the United States Naval War College, and director of the college's Civilian-Military Humanitarian Response Program.

^7^Director of Medical Operations for the USNS Comfort, during the 2010 earthquake response.

^8^This included case reports or case series.

^9^These domains were identified and developed during the preliminary analysis (structured interviews and key-report analysis); the initial abstract and subsequent full-text reviews did not establish any additional domains.

^10^Two-thirds of the team had existing experience, whilst one-third were junior staff, who had not been involved in previous disaster responses (29).

^11^At the peak of their operations, two months into the response, MSF were overseeing 26 individual facilities—one of these units, that was running throughout the entire response, was a fully functioning inflatable hospital (27).

^12^This included care delivered to patients who had presented with non-earthquake related pathology (27).

^13^Contributing organisations included Project Medishare, Healing Hands International, and the Haiti Hospital Appeal—who converted their specialist paediatric facility into an adult centre, with capacity for up to 22 patients (14).

^14^Such as education and agriculture sectors (25).

^15^Many did not speak Haitian Creole or French, and had not included trained interpreters as part of their response teams (19).

^16^Another component of the joint military command structure.

^17^At the time, there were only 10 psychiatrists in Haiti to serve the mental health needs of the entire country.

^18^It is worth noting that significant amounts of this funding remain unaccounted for, raising concerns that funds were inappropriately managed (86).

^19^For example, large USN ships, such as the USNS Comfort, are limited in how quickly they are able to arrive on-station, and as such, are not a “golden hour” asset. This should be used to further inform the anticipated case load, and the subsequent equipment and capabilities available.

^20^The World Health Organisation seems best placed to fulfil this role, given their experience.

^21^Notably, 90% of the Haitian population remain vulnerable to further disaster events (99).
